# Unraveling the molecular complexity: Wtap/Ythdf1 and Lcn2 in novel traumatic brain injury secondary injury mechanisms

**DOI:** 10.1007/s10565-024-09909-x

**Published:** 2024-08-07

**Authors:** Chaobang Ma, Caili Gou, Shiyu Sun, Junmin Wang, Xin Wei, Fei Xing, Na Xing, Jingjing Yuan, Zhongyu Wang

**Affiliations:** 1 Department of Anesthesiology, Pain and Perioperative Medicine, The First Affiliated Hospital of Zhengzhou University, No.1, Jianshe East Road, Erqi District, Zhengzhou, 450052 Henan China; 2 Henan Province International Joint Laboratory of Pain, Cognition and Emotion, Zhengzhou, 450052 Henan China; 3 Department of Human Anatomy Basic Medical College of Zhengzhou University, Zhengzhou, 450001 Henan China

**Keywords:** Traumatic brain injury, Lcn2, Wtap, Ythdf1, m6A modification

## Abstract

The primary aim of this research was to explore the functions of Wtap and Ythdf1 in regulating neuronal Lipocalin-2 (Lcn2) through m6A modification in traumatic brain injury (TBI). By employing transcriptome sequencing and enrichment analysis, we identified the Wtap/Ythdf1-mediated Lcn2 m6A modification pathway as crucial in TBI. In our in vitro experiments using primary cortical neurons, knockout of Wtap and Ythdf1 led to the inhibition of Lcn2 m6A modification, resulting in reduced neuronal death and inflammation. Furthermore, overexpression of Lcn2 in cortical neurons induced the activation of reactive astrocytes and M1-like microglial cells, causing neuronal apoptosis. In vivo experiments confirmed the activation of reactive astrocytes and microglial cells in TBI and importantly demonstrated that Wtap knockdown improved neuroinflammation and functional impairment. These findings underscore the significance of Wtap/Ythdf1-mediated Lcn2 regulation in TBI secondary injury and suggest potential therapeutic implications for combating TBI-induced neuroinflammation and neuronal damage.

**Supplementary Information:**

The online version contains supplementary material available at 10.1007/s10565-024-09909-x.

## Introduction

Traumatic brain injury (TBI) has emerged as a widely acknowledged and substantial neurological disorder in recent years, particularly affecting individuals involved in traffic accidents, workplace injuries, and military conflicts (Paget et al. 2021; Capizzi et al. 2020; Vella et al. 2017; Robinson 2021). TBI is not exclusively an immediate occurrence; its effects could persist for weeks, months, or even years, resulting in a substantial deterioration in the patient's quality of life (Martin et al. 2018; Stocchetti and Zanier 2016). Many studies are currently investigating TBI's intricate pathological mechanisms to discover more efficient treatment modalities (Zou et al. 2023; Wiles 2022). The activation of neurotoxic glial cells is crucial in developing secondary damage in TBI; however, the exact molecular regulatory mechanisms involved remain unclear (Au and Ma 2022).

M6A modification, being a type of RNA modification, has attracted considerable interest in recent times for its association with neurological disorders (Satterwhite and Mansfield 2022; Shi et al. 2020; Wang et al. 2021a; Xia et al. 2023). Specifically, investigating the interaction between m6A methyltransferases, recognition enzymes, and specific gene products in regulating cellular functions is a fascinating research area (Du et al. 2022). Lcn2 is a gene expressed in response to different physiological and pathological conditions. Recent studies have suggested its potential association with the stimulation of neurotoxic glial cells, yet the underlying mechanism of m6A modification requires further investigation (Javaid et al. 2023).

WTAP (Wilms tumor 1-associating protein) and YTHDF1 (YTH N(6)-methyladenosine RNA binding protein 1) are two crucial molecules that have a pivotal role in m6A modification (Jiang et al. 2021; Zhang et al. 2020; Wang et al. 2020). These proteins participate in the processes of m6A modification, both in writing and reading, by interacting with a range of gene products. This allows them to regulate the physiological functions of cells (Gray et al. 2022). The regulatory function of Wtap and Ythdf1 may be linked to neuronal injury, regeneration, and glial cell response, particularly within the nervous system (Claassen and Park 2022). Hence, investigating their role in mediating m6A modification of Lcn2, which in turn impacts the initiation of neurotoxic glial cell activation and the subsequent damage caused by TBI, holds immense scientific and clinical importance.

The focus of this study is to clarify the mechanism by which the m6A modification of Lcn2 in neurons, facilitated by Wtap and Ythdf1, controls the activation of neurotoxic astrocytes, thereby impacting the secondary injury in mouse TBI. Our objective is to identify new molecular targets for treating TBI and contribute to the recuperation of patients and enhanced quality of life through comprehensive mechanistic research.

## Materials and methods

### TBI mouse model construction

Prior to the experiment, 144 male C57BL/6 mice (213, Vital River, Beijing, China) were accommodated in facilities adhering to specific pathogen-free (SPF) animal facilities with consistent humidity levels of 45%-50% and temperatures ranging from 25–27 °C for one week. Mice underwent exposure to a light–dark cycle lasting 12 h each day to facilitate adaptation to the experimental conditions. Animal experimental procedures conducted in our institute have received approval from the Animal Ethics Committee.

Traumatic brain injury (TBI) results from the controlled cortical impact (CCI) on the brain's right hemisphere. Mice were anesthetized throughout the surgical procedure using the isoflurane vaporizer (901809, VetEquip, Inc., Livermore, CA) with a concentration of 2–2.5% isoflurane (PHR2874, Sigma-Aldrich, USA). The depth of anesthesia was monitored using the toe pinch reflex. Secure the mouse's head in a stereotactic frame and perform a midline incision, approximately 1 cm long, along the head to expose the skull. Preparation is needed for a right frontal craniotomy, with a planned center point of 0.5 mm anterior to the anterior fontanelle and 2.0 mm lateral. The diameter of the craniotomy should be 3.5 mm to adequately expose the dura mater and cerebral cortex. Cortical contusion injury (CCI) was induced using the PinPoint™ Precision Cortical Impactor (PCI3000-1, Hatteras Instruments Inc., USA). A tip with a 3 mm impact diameter applied vertical force to the brain's surface. The velocity upon impact is 3 m per second, with an impact span lasting 150 ms, and a penetration depth of 2 mm. Following a TBI, it is essential to promptly replace and secure the bone flap and subsequently suture the scalp. All aspects of the animals in the sham group, including anesthesia, skin incision, and craniotomy surgery, followed the protocol, except for CCI. During the surgery, it is advised to employ a heating pad to ensure that the temperature of the mouse's rectum remains at 37 ± 0.5 °C (Zhao et al. 2022; Ignowski et al. 2018; Krukowski et al. 2021; Wu et al. 2021a).

### Injection of lentiviruses and animal experiment grouping

The lentiviral expression systems were constructed by utilizing the lentiviral overexpression vector (LV4, GimaGen, Shanghai, China) and the lentiviral knockdown vector (pGLVU6/GFP, C06001, GimaGen, Shanghai, China). sh-Wtap (TRCN0000331380; target sequence: GGAAAGTACACAGATCTTAAT) and sh-NC(CTCGCTTGGGCGAGAGTAA) (Jin et al. 2017) The lentivirus was acquired from Sigma-Aldrich (USA) with a determined virus titer of 10^9^ TU/mL.

One week prior to TBI surgery, 2 μL of sh-Wtap (silencing Wtap lentivirus), sh-NC (silencing lentivirus negative control), oe-Lcn2 (overexpressing Lcn2 lentivirus), and oe-NC (overexpressing lentivirus negative control) lentiviruses were administered through injection into the right lateral ventricle of mice using a stereotaxic apparatus (SR-5R, Narishige Scientific Instruments Laboratory, Japan) with a flow rate set at 0.5 µL/min for 10 min. At a distance of 0.2 mm from the bregma, 1.0 mm away from the midline, and 1.5 mm beneath the brain's surface, the insertion point was precisely situated (Wu et al. 2021b, 2020; Lu et al. 2020).

The mice were grouped as follows: (1) Sham group, TBI group; (2) Sham + sh-NC + oe-NC group, TBI + sh-NC + oe-NC group, TBI + sh-Wtap + oe-NC group, and TBI + sh-Wtap + oe-Lcn2 group. Each group comprised 24 mice, out of which 8 mice were dedicated to neurobehavioral testing and assessment. After 3 days post-TBI surgery, 16 mice were euthanized, with the brains of 8 mice being employed for measuring brain water content and the remaining 8 mice's cortical tissue being utilized for subsequent histopathology, gene analysis, and protein detection.

### RNA retrieval and sequencing analysis

Isolation of total RNA was carried out from the ipsilateral cerebral cortex sections of mice in the Sham and TBI cohorts (3 days after surgery) using Trizol reagent (15596026, Invitrogen, Thermo Fisher Scientific, Waltham, MA, USA), with 4 samples per group. RNA sample concentration and purity levels were assessed with a Nanodrop2000 spectrophotometer (1011U, Nanodrop, Thermo Fisher Scientific, Waltham, MA, USA). Total RNA samples meeting the following criteria based on denaturing agarose gel electrophoresis and Bioanalyzer 2100 analysis for RNA integrity are used for subsequent trials: RNA integrity index (RIN) must be equal to or greater than seven (7.0) with a ratio of 28S to 18S exceeding 1.5.

CapitalBio Technology, located in Beijing, China, produced and sequenced the library for sequencing. Each sample utilizes a sum of 5 μg RNA. In summary, the Ribo-Zero™ Magnetic Kit (MRZE706, Epicentre Technologies, Madison, Wisconsin, USA) is employed to eliminate ribosomal RNA (rRNA) from total RNA. The NEB Next Ultra RNA Library Prep Kit (#E7775, NEB, USA), provided by NEB, USA, was utilized for Illumina sequencing to establish a repository dedicated to genetic sequencing. Next, the RNA fragments are broken down into fragments of about 300 base pairs (bp) using NEB Next First Strand Synthesis Reaction Buffer (5 ×). For the synthesis of the initial cDNA strand, a combination of reverse transcriptase primers and random primers was utilized, followed by the generation of the complementary cDNA strand in a reaction buffer comprising dUTP Mix (10 ×). Repairing the cDNA fragment involves adding a poly(A) tail and ligating a sequencing adaptor. Upon the joining of the Illumina sequencing adapters, the second cDNA strand underwent digestion using the USER enzyme (#M5508, NEB, USA) to generate libraries with strand specificity. Following this, the library DNA needs to undergo amplification, purification, and enrichment via PCR. The library was then assessed utilizing an Agilent 2100 system and quantified with the KAPA Library Quantification Kit (KK4844, KAPA Biosystems). Finally, the NextSeqCN500 (Illumina) sequencer was used to perform paired-end sequencing (Regan et al. 2021; Bao et al. 2023).

### Quality assessment of sequencing data and alignment with reference genome

The evaluation of the quality of paired-end reads within the initial sequencing dataset was conducted using FastQC v0.11.8 software. The initial data underwent preprocessing via Cutadapt software version 1.18 to eliminate Illumina sequencing adapters and poly(A) terminal sequences. Eliminate reads with an N content surpassing 5% by employing a Perl script. Seventy percent of the total reads were retained after extracting those with a base quality score exceeding 20. It was done using version 0.0.13 of the FASTX Toolkit software. The paired-end sequences were corrected utilizing BBMap software. Afterwards, the screened and superior read fragments were mapped to the mouse reference genome employing hisat2 software (version 0.7.12) (Bao et al. 2023).

### Bioinformatics analysis

We obtained the transcriptome sequencing dataset GSE167459 from the GEO database (http://www.ncbi.nlm.nih.gov/geo/). This dataset is composed of TBI-associated microglial and astrocytic cells. It consists of 5 samples from both sham and TBI groups for microglial cells and 5 samples from both sham and TBI groups for astrocytic cells. Differential expression analysis was conducted on the sequencing data and dataset GSE167459 utilizing the "edgeR" package in R language (version 4.2.1), based on mRNA read count. The criteria for differential gene selection were set as |logFC|> 1 and P.value < 0.05. The generation of volcano plots and boxplots for differentially expressed genes could be accomplished using the "ggplot2" package in R, while a heatmap of differential gene expression could be created using the "heatmap" package in R. After converting the obtained differentially expressed genes into ID format, we will perform enrichment analysis using the "clusterProfiler" package in R software. The z-score values for each enriched term will be calculated using the provided molecular values with the GOplot package. Finally, a bar plot depicting the enrichment analysis results will be created (Yu et al. 2012; Walter et al. 2015). The STRING database (https://string-db.org/) analyzes protein–protein interactions encoded by genes. It conducts network analysis on the top 20 genes that encode proteins with differences and measures their degree of centrality. Additionally, we performed further extraction and analysis of the expression statuses of factors associated with m6A modification from the transcriptome sequencing results. Subsequently, Spearman correlation analysis was performed on the highest-ranked genes by their centrality and the m6A regulators Wtap and Ythdf1. We utilized the SRAMP website to predict the m6A binding sites on the target gene Lcn2 RNA and generated a schematic diagram illustrating the secondary structure of the m6A site (Zhou et al. 2016).

### Neuropsychological testing and assessment

Two research personnel who were unaware of the experimental design assessed neurological deficits. The assessment included baseline, TBI, and sham surgery through a comprehensive modified neurological severity score (mNSS), the rotarod test, and the footprint analysis on days 1, 3, 7, and 14. Every behavior is tested using four experiments, repeated twice to ensure data verification.

The mNSS score could be used to assess several key aspects of motor function in mice, including muscle condition, abnormal movement, sensory function (vision, touch, proprioception), equilibrium, and reflex capabilities. Neurological function is assessed on a scale ranging from 0 to 18, where a rating of 0 signifies typical function while a rating of 18 denotes the utmost degree of dysfunction. Normal mice were assigned a score of 0 points. An mNSS score between 1 and 6 was classified as mild injury, while a score ranging from 7 to 12 was classified as moderate injury, and a score with a range of 13 to 18 was classified as severe injury (Xu et al. 2017; Chen et al. 2018).

Using the Rota-Rod treadmill (LE8205, Shenzhen Rewood Life Technology Co., Ltd), the Rotarod test is employed to assess motor coordination and limb strength. The testing process comprises six-speed change experiments. These experiments begin with an initial speed of 5 rpm for the first 10 s, followed by a gradual rise from 5 to 10 rpm over the subsequent 30 s. Finally, the speed linearly increases from 10 to 20 rpm between 40 and 90 s. The experiments initiate with an initial velocity of 5 rThe ultimate score is calculated according to the mean duration that the mice were able to sustain their equilibrium on the rod across the six trials (Wu et al. 2021a).

Foot fault test: Evaluating the functionality of motion. Place the mice on hexagonal grids of various dimensions. The mouse places its paws on the wire as it traverses the grid. While carrying a load with each step, the claws may land or slide between the metal wires. A foot fault has been documented. Determine the total count of steps the mouse takes to traverse the grid, considering the movement of each forelimb while also monitoring the overall number of errors in foot placement for every forelimb. Determine the ratio of forelimb mistakes to the overall steps (Wu et al. 2021a).

### Morris water maze test (MWM)

The MWM was carried out to measure mice's spatial cognitive and mnemonic capabilities on days 15–21 after TBI. The MWM pool comprises a stainless steel cylindrical structure measuring 122 cm in diameter and 51 cm in height. This structure also includes an underwater concealed platform with a diameter of 10 cm. The MWM device is filled with water at 22 ± 2 °C and colored white using non-toxic paint. The experiment comprises two sequential stages: the training phase, which spans from day 15 to day 20, and the spatial memory testing phase on day 21. Four latency tests are conducted daily during the training phase, each lasting 90 s. The mice (*n* = 8/group) distributed among the quadrants of the pool, starting from the first quadrant and ending at the fourth quadrant. The time a mouse locates the platform within a 90-s timeframe and remains on it for 5 s is documented as the escape latency period. If the mouse fails to locate the platform within 90 s, it is then directed to the platform and given a 15-s period to stay on it. The time taken for the mouse to escape is recorded as 90 s. In the testing phase, after removing the platform, the mice were then relocated to the quadrant positioned diagonally across from the platform, where they were given 90 s to search for its location. The video tracking system (EthoVision XT 13, Noldus Information Technology, Wageningen, Netherlands) was employed for recording and analyzing platform crossing time, escape latency, and swimming trajectory (Liu et al. 2023; Zhao et al. 2022).

### Measurement of brain water content

By the third day after TBI, the mice were humanely put down, and their brains were harvested without undergoing cardiac perfusion. The injured hemisphere tissue sample was dissected and measured using an electronic analytical scale to ascertain its wet weight. The dry weight was obtained by drying it at 100 °C for 48 h. The mathematical expression to determine brain water content is provided as follows: Brain water content (%) = (wet weight—dry weight) / wet weight × 100% (Chen et al. 2018).

### Volume assessment of contusion

To quantify the extent of contusion located in the cortex on the same side three days after TBI, the sections that were stained underwent digitization and analysis through ImageJ software after being treated with cresyl violet. To calculate the volume, the area of the damage should be added together and multiplied by the interlayer distance (500 μm). The percentage loss in hemispherical organization could be calculated using the subsequent equation:

(Volume of the opposite hemisphere—Volume of the same hemisphere)/(Volume of the opposite hemisphere) × 100% (Wu et al. 2021a).

#### ELISA

Weigh 0.1 g of cortical tissue from each group of mice and transfer it to 900 μL of saline solution. Subsequently, sonicate and centrifuge the mixture at 3000 g for 15 min to obtain a tissue homogenate. The total protein concentration in the tissue homogenate was measured using the BCA Protein Assay Kit (P0012S, Beyotime, Shanghai, China). Additionally, the cell culture medium and supernatant from primary cortical neurons were collected for each group. The levels of interleukin-1β (IL-1β; PI301, Beyotime, Shanghai, China), IL-6 (PI326, Beyotime, Shanghai, China), and tumor necrosis factor α (TNF-α; PT512, Beyotime, Shanghai, China) were measured in the tissue homogenate and cell culture supernatant following the instructions provided by the ELISA kits. Absorbance values were assessed at 450 nm using an enzyme-linked immunosorbent assay (ELISA) reader (Bio-Tek, Winooski, VT, USA). Analyze using Origin 9.5 software (Cui et al. 2018; Xu et al. 2017).

### Immunofluorescent staining

Three days after TBI, the mice are anesthetized, and cardiac perfusion is conducted using 0.9% NaCl. Subsequently, the injured brain tissue is dissected. Subsequently, we will utilize a paraformaldehyde solution with a concentration of over 4% to fix the brain tissue. We will then transfer it to a temperature-controlled environment at 4 °C, where it will be immersed in a 25% glucose phosphate buffer to facilitate dehydration. Once dehydration is complete, the tissue will be carefully removed, and the surface moisture will be dried. Subsequently, a surgical knife will flatten the targeted tissue before placing it on a specimen tray. Subsequently, we will employ an OCT embedding medium to envelop the tissue and position the specimen tray on the freezing stage of the cryostat for prompt freezing and embedding. We could begin slicing once the OCT has turned white and hardened. Our process involves cutting continuous sagittal sections with an approximate thickness of 10 μm. Following sectioning, the slices will be rinsed with room temperature phosphate-buffered saline (PBS), with each rinse lasting 5 min. This rinsing process will be repeated a total of three times. Next, the tissue sections will undergo permeabilization and blocking with a solution of 1% donkey serum containing 0.3% Triton X-100 for one hour. Next, the brain tissue will be incubated overnight with a primary antibody at 4 °C. Following overnight incubation, the frozen sections will undergo a 1-h incubation at room temperature using the following secondary antibodies: goat anti-rabbit (Cy3 ®; 1:1000, ab6939, Abcam, UK), goat anti-mouse (Alexa Fluor® 488; 1:1000, ab150117, Abcam, UK), and goat anti-rat (Alexa Fluor® 488; 1:1000, ab150157, Abcam, UK). In the future, the slices will be washed using PBS and stained with DAPI. The detailed information for one of the antibodies is as follows: Rabbit anti-Iba-1 (1:200, ab178846, Abcam, UK) or Mouse anti-Iba-1 (1:100, ab283319, Abcam, UK), Mouse anti-GFAP (1:50, ab4648, Abcam, UK), Rabbit anti-C3 (1:100, ab97462, Abcam, UK), Rat anti-CD16/32 (1:200, ab25235, Abcam, UK), Rat anti-CD206 (1:100, MA5-16,871, Thermo Fisher Scientific, Waltham, MA, USA), Mouse anti-NeuN (1:100, ab104224, Abcam, UK), and Rabbit anti-Lcn2 (1:200, A2092, Abclonal, Wuhan, China) (Wang et al. 2021b; Chen et al. 2018).

We collected coronal slices ranging from -1.0 to -3.0 mm of the anterior fontanelle for immunofluorescence image analysis and quantification. We randomly selected five non-adjacent sections spaced 100 μm apart in each animal. Subsequently, we choose five regions of interest (ROIs) from these sections for further analysis. These regions of interest (ROIs) are approximately 200 μm adjacent to the boundary of brain lesions. ImageJ software is utilized to analyze the fluorescence intensity and determine the ratio of Iba-1 + , GFAP + C3 + , Iba-1 + CD16/32 + , Iba-1 + CD206 + , Iba-1 + Lcn2 + , GFAP + Lcn2 + , and NeuN + Lcn2 + cells (Wu et al. 2021a).

### TUNEL staining detects neuronal apoptosis in tissues

To better evaluate neuronal apoptosis in the traumatized cortical region, we utilized the immunofluorescent double staining technique to label terminal deoxynucleotidyl transferase dUTP nick-end labeling (TUNEL) and neuronal nuclear (NeuN). This allowed us to investigate the proximity of apoptotic cells to neurons in a given area. Immunostaining of frozen sections was conducted using a rabbit anti-NeuN antibody (1:100, ab190195, Abcam, UK) overnight at 4 °C. Subsequently, TUNEL staining was conducted through the in situ cell death detection kit from Beyotime (C1089, Shanghai, China). Finally, the slices were covered with DAPI (C1006, Beyotime, Shanghai, China). A confocal laser scanning microscope (CLSM; LSM 510 META, Carl Zeiss AG) was utilized to observe and quantify six arbitrary microscopic fields per section. Three sections were analyzed each individual animal. All counting is done in Braille. The findings are presented as the proportion of total neurons experiencing apoptosis, as determined through dual staining with NeuN and TUNEL/NeuN stained cells (Xu et al. 2017).

### Quantification of RNA m6A methylation

The procurement of overall RNA from tissues and cells was executed with the application of TRIzol reagent as directed by the manufacturer. The global quantities of m6A RNA were observed and recorded using the EpiQuik m6A RNA Methylation Quantification Kit (ab185912, Abcam, UK). Apply 200 ng of purified PolyA + mRNA onto the assay well. Next, the total RNA was immobilized onto the strip wells using a high-affinity RNA solution. Antibodies for capturing and detecting m6A levels are included in the reagent kit. Measure the light absorption of individual RNA specimens at 450 nm with a microplate spectrophotometer, followed by colorimetric quantification to determine the m6A level (Shen et al. 2021; Wang et al. 2023a).

### m6A dot plot experiment

Mix the cellular RNA and the organism RNA with an equal volume of 20 × SSC buffer (S6639, Sigma-Aldrich, USA). Incubate the mixture at 65 °C for 5 min to induce denaturation. Next, add 100 ng, 200 ng, or 400 ng of poly(A) + RNA onto the Hybond N + membrane (YA1760, Solarbio, Beijing, China). After exposing the membrane to UV crosslinking for 30 min, rinse it with PBST buffer and then block it using 5% non-fat milk. Next, the membrane went through incubation for the duration of the night at 4 °C with an anti-m6A antibody (mouse, 1:1000, 68,055–1-Ig, Proteintech, Wuhan, China). Next, incubate the sample with the secondary antibody at the ambient conditions for one hour and visualize the result using enhanced chemiluminescence (ECL). Apply an equal quantity of RNA onto the membrane and then stain it with a 0.02% methylene blue solution for 2 h (Shen et al. 2021; Song et al. 2019).

### MeRIP-qPCR

The total RNA (100 µg) extracted from cells and tissues was then incubated with either anti-m6A antibody (mouse, 68055–1-Ig, 1:1000, Proteintech, Wuhan, China) or anti-IgG (Rabbit, 1:100, 30,=000–0-AP, Proteintech, Wuhan, China) coupled to protein A/G magnetic beads in IP buffer (140 mM NaCl, 1% NP-40, 2 mM EDTA, 20 mM Tris pH 7.5; enriched with protease inhibitors and RNase inhibitors) overnight at 4 °C. Incubate 300 μL of elution buffer containing 0.05% SDS, 1 mM EDTA, and 5 mM Tris–HCl with 8.4 μg of proteinase K (9034, Takara, Beijing, China) held at 50 °C for a time span of 1.5 h. Subsequently, remove RNA from the beads. Quantification of m6A modification levels in Lcn2 was achieved through RT-qPCR (Shen et al. 2021; Wang et al. 2023a).

### RIP PCR experiment

Mutant plasmids for Lcn2 were constructed by introducing mutations at the m6A site using PCR-based methods. The coding sequence (CDS) for synthesizing Lcn2 mRNA should be cloned into the vector pCDNA3.1 (V79520, Invitrogen, ThermoFisher Scientific, USA). Furthermore, the adenine (A) at position 442 and position 507 were mutated to guanine (G) to construct the mutant plasmids Lcn2-MUT1 and Lcn2-MUT2 of Lcn2.

Based on the experimental protocol, RIP assays were conducted using the EZ-Magna RIP™ RNA-binding protein immunoprecipitation kit (17–701, Millipore, Sigma-Aldrich, Billerica, MA, USA). We used RIP lysis buffer containing RNase and protease inhibitors to lyse 90% of confluent cells from each group. The RNA extract (100 µl) was incubated with either an anti-Ythdf1 antibody (rabbit; 1:100, 17479–1-AP, Proteintech, Wuhan Sanying, China) or an anti-Wtap antibody (rabbit; 1:100, 10200–1-AP, Proteintech, Wuhan Sanying, China) conjugated magnetic beads in the RIP buffer. A negative control, Anti-IgG (Rabbit, 1:100, 30000–0-AP, Proteintech, Wuhan Sanying, China), was utilized (Wang et al. 2023a).

### RNA stability experiment

Actinomycin D (Act-D; 5 µg/ml; A9415, Sigma-Aldrich, USA) could be employed to assess RNA stability in cells. Cells should be harvested, and RNA should be extracted at specific time points after incubation for RT-qPCR analysis (Wang et al. 2023a).

### Isolation, cultivation and identification of primary cells

Primary cortical neurons were isolated from wild-type C57BL6 mice on postnatal days 1–3. First, methodically disconnect the meninges and vascular structures to achieve a distinct separation of the cerebral cortex. Next, the tissue was finely diced and digested using 0.25% trypsin (25200–072, Thermo Fisher Scientific, Waltham, MA, USA) at 37℃ for 20 min, with subsequent gentle agitation. The dispersed cells were individually seeded onto 6-well plates, 96-well plates, and coverslips (24 mm × 24 mm) coated with poly-L-lysine (100 μg/ml). They were cultivated in Neurobasal Medium Plus (A3582901, Thermo Fisher Scientific, Waltham, MA, USA), supplemented with 2% B27 supplement (A3582801, Thermo Fisher Scientific, Waltham, MA, USA), 0.5 mM L-glutamine, and 50 U/ml penicillin/streptomycin. The cultures were maintained in a 5% CO_2_ atmosphere at 37 °C. Replace the culture medium every 8 h and then exchange half every other day. Experiments using neuronal cultures are conducted 8 to 10 days after they are seeded (Cai et al. 2022; Lai et al. 2020; Xing et al. 2014).

Primary microglia were isolated from wild-type C57BL6 mice on postnatal days 1–3 after birth. The meninges were removed, and the cortical tissue was digested at 37 °C for 30 min using 0.25% trypsin–EDTA. Subsequently, the tissue was mechanically triturated in DMEM/F12 medium (12400–024, Thermo Fisher Scientific, Waltham, MA, USA) supplemented with 10% fetal bovine serum (FBS) (26140079, Thermo Fisher Scientific, Waltham, MA, USA). Mixed dermal cells should be inoculated in DMEM/F12 culture medium supplemented with 10% FBS. The culture medium should be completely replaced every 3–4 days to ensure optimal growth. Confluence should be achieved after approximately 10–12 days *in vitro*. After 15–18 days of incubation, the mixed neuroglial cell cultures were treated with a trypsin solution (0.25% trypsin–EDTA diluted 1:4 in DMEM/F12) for 15–25 min. This treatment detached the intact cell layer, while the small glial cells stayed adhered to the flask's base, isolating the small glial cells.

Primary astrocytes were separated from wild-type C57BL6 mice between postnatal days 1 and 3. In brief, astrocytes were obtained from a mixed glial culture of cortical cells after 10–14 days of culture. This was achieved by shaking the culture overnight at 220 g to eliminate microglia and oligodendrocytes. The star-shaped glial cells underwent digestion and dissociation using trypsin before being re-seeded onto a cell culture plate coated with collagen proteins. Experiments were conducted using primary cultured astrocytes from the 2nd to the 4th generations.

Identification: Immunofluorescence staining was used to identify primary cortical neurons, small glial cells, and astrocytes. The cells were immobilized in a 4% paraformaldehyde solution at ambient temperature for 30 min. Following PBS washing, a blocking solution (consisting of 5% donkey serum and 0.1% Triton X-100 in PBS) was applied to the cells and left to incubate at room temperature for 2 h. 100 μL of rabbit anti-III class β-tubulin (1:100 dilution, ab18207, Abcam, UK), rabbit anti-Iba-1 (1:200 dilution, ab178846, Abcam, UK), and rabbit anti-GFAP (1:5000 dilution, ab7260, Abcam, UK) were then added to coverslips. Over a night-long period at 4 °C, cells were incubated with the coverslips. Then, depending on the primary antibody selected, incubate the samples with either Goat Anti-Rabbit Secondary Antibody (Cy3®; 1:1000, ab6939, Abcam, UK) or (Alexa Fluor® 488; 1:1000, ab150077, Abcam, UK) for 1 h at room temperature. Finally, cell nuclei were labeled with DAPI and then observed and imaged using confocal fluorescence microscopy (Rangaraju et al. 2018; Zhu et al. 2022; Zhong et al. 2021; Kang et al. 2018; Xing et al. 2014). The specificity and purity of the cultured microglial cells were assessed utilizing flow cytometry. The cells were labeled with anti-CD11b-FITC antibody (11–0112-82, Thermo Fisher Scientific, Waltham, MA, USA) for fluorescence detection, and over 95% of the cells were found to be CD11b positive, indicating a high level of purity in the cultured microglial cells (Xing et al. 2014).

### *in vitro* TBI model

Developing an *in vitro* TBI model through mechanical stretch-induced injury. Prior to a 5-day stretch exposure, primary cortical neurons (7 × 100,000 cells/well) were seeded onto a silicone membrane in custom stainless steel wells. The hole should be installed into the stretching device and subjected to severe stretching damage, simulating a strain field model similar to TBI in human bodies. The strain rate should be set at 10/s, the membrane deformation at 50%, and the peak pressure at 3–4 psi (Kenny et al. 2019; Bao et al. 2021; Ji et al. 2012).

### Cell death assessment

The assessment of cell cytotoxicity employed the lactate dehydrogenase (LDH) assay kit (MAK066-1, Sigma-Aldrich, USA). Cortical neurons were cultured in 6-well plates at 1 × 10^6^ cells per well for 7 days. After processing, collect 75 μl of supernatant from each sample. Then, react the collected supernatant with 150 μl of LDH reagent at room temperature for a period of 20 min. Utilizing a microplate reader, the sample's optical density (OD) was gauged at 490 nm. In the above statement, the term 'release of LDH' pertains to the percentage of LDH present in the supernatant relative to the total LDH (which includes intracellular and supernatant LDH) (Xu et al. 2021).

### Neurotoxicity-induced oligodendrocytes and astrocytes

Neurotoxicity in microglial cells was induced by lipopolysaccharide (LPS, 1 μg/ml; Sigma Aldrich, USA) and interferon-gamma (IFN-γ, 20 ng/ml; Sigma Aldrich, USA). Astrocytes exhibit neurotoxicity when induced by C1q (400 ng/ml; MCE, USA), TNF-α (30 ng/ml; Abcam, UK), and IL-1α (3 ng/ml; Abcam, UK) (Wang et al. 2021b). To assess the neurotoxicity of induced microglial cells and astrocytes, the cells were stimulated for 24 h. Subsequently, the supernatant from the culture medium was collected to obtain microglial cell-conditioned medium (MCM) and astrocyte-conditioned medium (ACM). Then, replace half of the original cortical neuron culture medium with either MCM or ACM. Incubate the cells for 24 h to measure the release of lactate dehydrogenase (LDH) and apoptosis rate. The experimental groups consisted of microglia treated with either PBS or LPS + IFN-γ, astrocytes treated with PBS or C1q + TNFα + IL-1α, and primary cortical neurons treated with untreat-M (uninduced microglial cell culture medium), MCM, untreat-A (uninduced astrocyte culture medium), and ACM.

### Cell apoptosis detection

Cell apoptosis was detected using the Flow Cel Assay Kit (APOAF, Sigma Aldrich, USA). Prepare a suspension of primary cortical neurons (1 × 10^5^/mL). Next, sequentially add 5 µL of FITC-Annexin V and PI. Next, incubate the sample in a dimly lit room at ambient temperature for a duration of 20 min. The Guava® easyCyte™ 6-2L Base System flow cytometer (0500–5007, Luminex) was used to detect cellular apoptosis. Perform data analysis using CellQuest Pro software. Apoptotic cells contribute to Quadrants 2 and 3 (Lu et al. 2020; Zhao et al. 2018).

### CRISPR/Cas9

We utilized a transposon plasmid to induce deletions in the Wtap and Ythdf1 genes using the CRISPR/Cas9 system. This transposon plasmid consists of an expression cassette for the spCas9 enzyme, a gene for purine resistance, and a chimeric guide RNA. Separately, the sgRNAs targeting the Wtap and Ythdf1 genes were designed using an online website and subsequently synthesized by Shanghai Biotech, a company based in China. Ythdf1-sgRNA1: 5'-CGTGACAGGCCCGGCCTCGTGGG-3'; Ythdf1-sgRNA2: 5'-TGGACTACGGCACCAGCGCTGGG-3'; Wtap-sgRNA1: 5'-GCTGCGAGACCCGCAAATAAAGG-3'; Wtap-sgRNA2: 5'-ATGGCAGCTCCTCCCGCCAGAGG-3'. Transfecting cells using Lipofectamine 3000 (Invitrogen, Thermo Fisher, USA). The transfected cells were selected 24 h after transfection using puromycin (2 µg/mL) from MCE, USA. After three days, transfer the individual bacterial colony into a 96-well plate. Genomic DNA extraction was conducted using the Quick-DNA Miniprep Kit (Zymo Research) for identification of deletions or insertions in the target colonies Wtap and Ythdf1. Sanger sequencing (GENEWIZ, China) was conducted on 5–10 bacterial colonies to acquire plasmids, followed by Chromas software sequence analysis. Select positive clones for downstream research. All clones are maintained in an identical environment to the parent cell (Lin et al. 2023).

### Plasmid transfection and cell experiment grouping

The Lcn2 gene should be ligated with the eukaryotic expression vector pCDNA3.1 to generate the overexpression plasmid of Lcn2, called oe-Lcn2. Primary cortical neurons were plated with a density of 2 × 10^5^ cells per well in a 6-well plate and maintained in a CO_2_ incubator for 24 h. Following this, the target plasmid was transfected into the primary cortical neurons using Lipofectamine 3000. According to the instruction manual for the reference reagent, the recommended concentration for plasmid usage is 50 nM. However, adjustments should be made based on specific circumstances. The cells were cultured at 37℃ and 5% CO_2_ after transfection for 6 to 8 h. Subsequently, after complete medium replacement, a culture period of 48 h was allocated for the cells. RNA and protein extraction was conducted for further investigations (Sato et al. 2010).

The specific cell experiment groups consist of the following: primary cortical neurons, (1) control group, Model group (*in vitro* TBI model group); (2) TBI model primary cortical neurons: vector group (plasmid vector control), Wtap-KO group (Wtap gene knockout), Ythdf1-KO group (Ythdf1 gene knockout); (3) TBI model primary cortical neurons: vector + oe-NC group, Wtap-KO + oe-NC group, Wtap-KO + oe-Lcn2 group, Ythdf1-KO + oe-NC group, Ythdf1-KO + oe-Lcn2 group; primary astrocytes and microglia: (1) control group, Model group, Model + oe-NC group, Model + oe-Lcn2 group. In each treatment group, the conditioned medium obtained from the primary cortical neuron culture is added to the primary astrocytes and microglia (replacing half of the original culture medium) for co-culture for 24 h (Yang et al. 2020; Ye et al. 2020).

### RT-qPCR

Trizol reagent was employed for the extraction of total RNA from tissues and cells, followed by assessment of RNA concentration and purity using a Nanodrop 2000 spectrophotometer. Reverse transcription of RNA into cDNA was performed complying with the directives given in the PrimeScript RT reagent Kit (RR047A, Takara, Japan). The reverse transcription conditions are 42 °C for 30–50 min and then 85 °C for 5 s. We employed the Fast SYBR Green PCR Kit (RR820A, Takara, Japan) and ABI PRISM 7300 RT-PCR System (Applied Biosystems) for RT-qPCR detection. Details of the reaction conditions were as follows: pre-denaturation at 95 °C for 5 min, denaturation at 95 °C for 30 s, annealing at 57 °C for 30 s, extension at 72 °C for 30 s, for a total of 40 cycles. Each hole is equipped with three duplicates. β-actin serves as an internal control, and the relative gene expression levels are assessed employing the 2^−ΔΔCt^ method. Here, ΔΔCt is computed through the disparity between the average Ct value of the target gene in the experimental group and the average Ct value of the reference gene in the experimental group minus the difference between the average Ct value of the target gene in the control group and the average Ct value of the reference gene in the control group (Jin et al. 2019). Iterations of the experiment were performed in triplicate. The primer sequences have been listed in Table S1.

### Western Blot

Different groups of cells and tissues were collected and added separately to RIPA lysis buffer (P0013B, Beyotime, Shanghai, China) containing 1% PMSF on ice. The lysis process was performed for 30 min at 14,000 g and 4 ℃. Following centrifugation, the supernatant was collected. The protein extract was assessed for its protein concentration using the BCA method (P0012S, Beyotime, Shanghai, China). Add the appropriate amount of 5 × loading buffer to denature the proteins and boil at 100 °C for 10 min. The protein sample amount is 50 μg. The gel should be separated using separation and concentration gels specifically designed for electrophoresis. Following electrophoresis, the bands containing the desired protein should be transferred onto a PVDF membrane. The PVDF membrane was then incubated in 5% skim milk powder and sealed at room temperature for 1 h. Primary antibodies, including anti-Wtap antibody (mouse, 1:5000, 60188–1-Ig, Proteintech, Wuhan, China) or anti-Ythdf1 antibody (rabbit, 1:1000, 17479–1-AP, Proteintech, Wuhan, China), anti-Lcn2 antibody (rabbit, 1:500, A2092, Abclonal, Wuhan, China), anti-cleaved caspase-3 antibody (rabbit, 1:1000, #9661, Cell Signaling Technology, USA), anti-Bax antibody (rabbit, 1:1000, #2772, Cell Signaling Technology, USA), anti-Bcl-2 antibody (rabbit, 1:1000, #3498, Cell Signaling Technology, USA), and rabbit anti-β-actin (1:1000, ab8226, Abcam, UK) were introduced and allowed to incubate overnight at 4 °C. The internal control function was fulfilled by β-actin. Following incubation, the membranes were washed with phosphate-buffered saline with Tween (PBST) at room temperature and subsequently incubated with goat anti-rabbit/goat anti-mouse IgG (HRP) secondary antibodies (1:10000, BA1054/BA1050, Bioss, Wuhan, China) at room temperature for 1 h. Subsequently, the membranes underwent 6 rounds of washing with PBST for 5 min each. Evenly distribute the ECL reaction solution (AR1172, BioTek, Wuhan, China) onto the membrane. Subsequently, position the membrane in the imaging instrument (Amersham Imager 600, USA) for exposure. Then use Image J for grayscale analysis (Salem et al. 2019; Shu et al. 2011). The experiment is retested thrice to ensure accuracy.

### Statistical analysis

The research data was subjected to statistical analysis through the use of SPSS software (version 21.0, IBM, USA). The data measurements are expressed using Mean ± standard deviation. Initially, normality and homogeneity of variance tests are conducted. If the data show a normal distribution and have homogeneity of variance, a non-paired t-test is employed for between-group comparisons. For comparing multiple groups, one-way ANOVA or repeated measures ANOVA is used, and post hoc assessments are carried out with Tukey's technique. Correlation analysis between the target genes and their m6A regulatory factors was conducted using Spearman's method (Velazquez et al. 2019; Wang et al. 2023b; Tian and Yuan 2018). A significance level below 0.05 implies the existence of a statistically significant difference.

## Results

### Comprehensive analysis of transcriptome changes and inflammatory pathway activation in a mouse model of traumatic brain injury (TBI)

TBI is a profoundly debilitating neurological disorder and remains a prevalent factor contributing to global mortality and disability (Wu et al. 2021a). This factor risks developing chronic neurological disorders, including epilepsy, stroke, mental health disorders, and neurodegenerative illnesses (Wilson et al. 2017).

A TBI mouse model was initially established to examine the mechanisms underlying TBI. Figure S1A displays representative whole-brain images of the Sham and TBI groups taken at 1, 3, 7, and 14 days after the surgery. The TBI mice displayed brain injury on days 1 and 3 following the surgery, gradually ameliorating by days 7 and 14. Subsequently, neurobehavioral tests and evaluations were conducted on each set of mice. The TBI group of mice exhibited a substantial rise in mNSS scores and hindlimb motor dysfunction in contrast to the Sham group.

Moreover, in the rotarod assessment, the TBI group exhibited a reduced time to fall than the Sham group (Figure S1B-D). The experimental results of the MWM indicated that the TBI group of mice exhibited a longer escape latency as opposed to the Sham group. Moreover, the duration for the TBI group mice to navigate the platform was noticeably shortened. However, no variance was observed in swimming speed among the two mouse cohorts (Figure S1E). The swimming trajectory is depicted in Figure S1F.

Furthermore, we evaluated the volume of brain contusion and the presence of tissue edema in both the Sham and TBI groups at 3 days following the injury. The results revealed damage within the brain tissue of mice in the TBI cohort, accompanied by a marked increase in brain edema in the ipsilateral hemisphere (Figure S1G-H). The results indicate the effective development of a mouse model to replicate TBI.

**Fig. 1:**
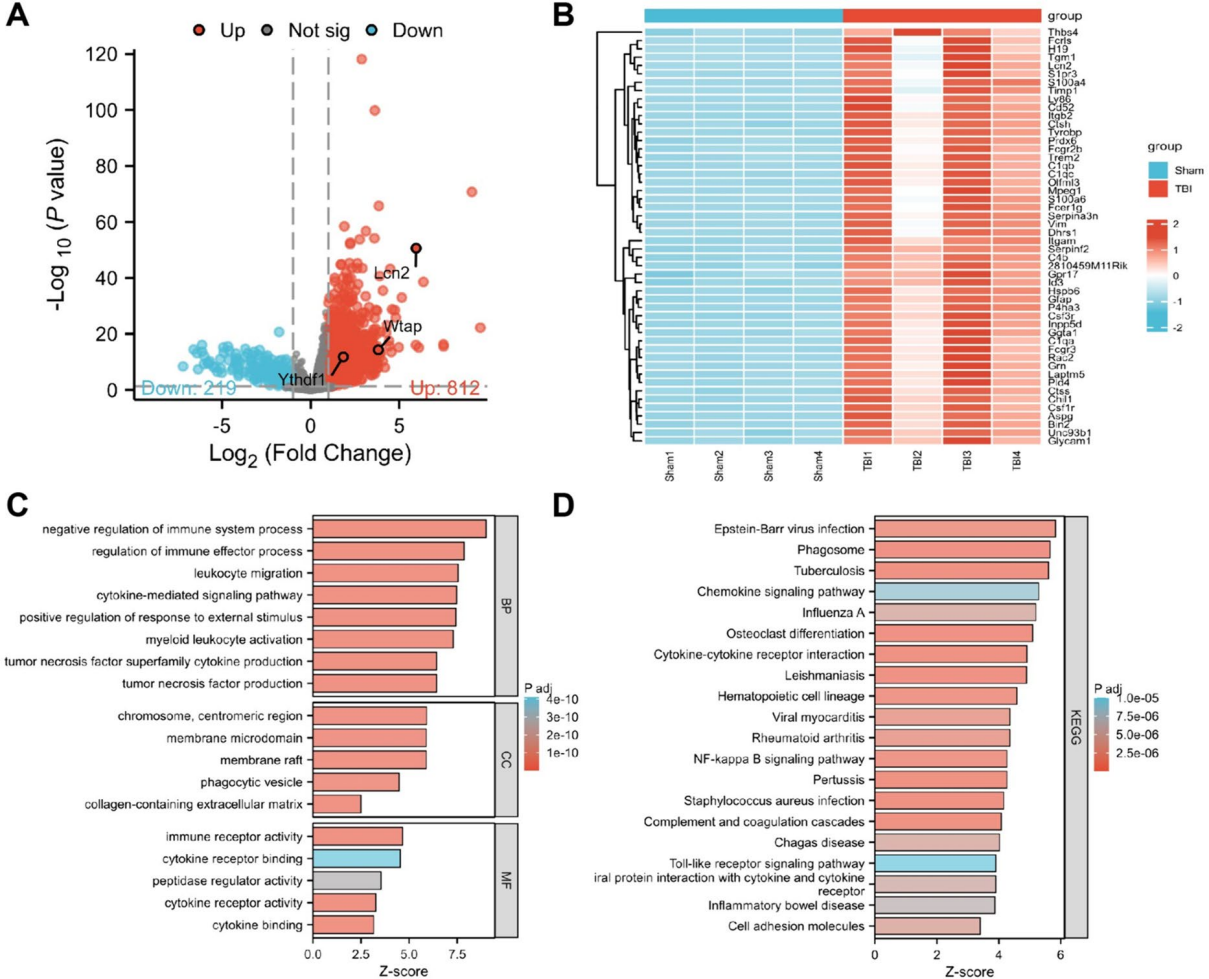
Analysis of differentially expressed genes and enrichment analysis of differential genes in transcriptional sequencing. (**A**) Volcano plot showing the differential gene expression in mouse brain tissue between the Sham and TBI groups; Sham group: *n* = 4, TBI group: *n* = 4; (**B**) Heatmap displaying the differential expression of the top 50 genes in mouse brain tissue between the Sham and TBI groups; Sham group: *n* = 4, TBI group: *n* = 4; (**C**) Enrichment analysis results of the differential genes in GO functions; (**D**) Enrichment analysis results of the differential genes in KEGG pathways. The X-axis in C-D represents the Z-score, and the Z-score indicates whether the corresponding entry is positively regulated (positive Z-score) or negatively regulated (negative Z-score); the color represents significance, with red indicating a greater difference

Subsequently, we obtained cerebral cortex tissues from mice in the Sham and TBI groups for transcriptome sequencing analysis. Volcano plots and heat maps displaying differential gene expression are presented in Fig. 1A-B. A total of 1031 genes have been found to exhibit differential expression, including 812 genes with increased expression and 219 genes showing decreased expression levels. GO and KEGG enrichment analyses were conducted on all differentially expressed genes. The inferences drawn from the GO functional analysis pointed out that the differentially expressed genes primarily regulated the immune system and immune processes, cytokine-mediated signaling pathways, and tumor necrosis factor production, among other biological processes (BP). Cellular components (CC) were primarily concentrated in the extracellular matrix containing collagen, chromosome centromeric regions, membrane rafts, membrane microdomains, and other terms.

Regarding molecular function (MF), they mainly participated in cytokine or cytokine receptor binding, highlighting functions in immune receptor activity and peptide enzyme regulatory activity (Fig. 1C). The results of the KEGG pathway enrichment analysis revealed that the differentially expressed genes were primarily enriched in several signaling pathways, including the Chemokine signaling pathway, Cytokine-cytokine receptor interaction, NF-kappa B signaling pathway, Complement and coagulation cascades, and Toll-like receptor signaling pathway (Fig. 1D). These results suggest that cytokines and inflammation-related pathways are pivotal in controlling the development and course of TBI.

### Exploring the role of m6A modification in the regulation of key genes and neuroinflammation in TBI: a transcriptomic and protein interaction analysis

**Fig. 2:**
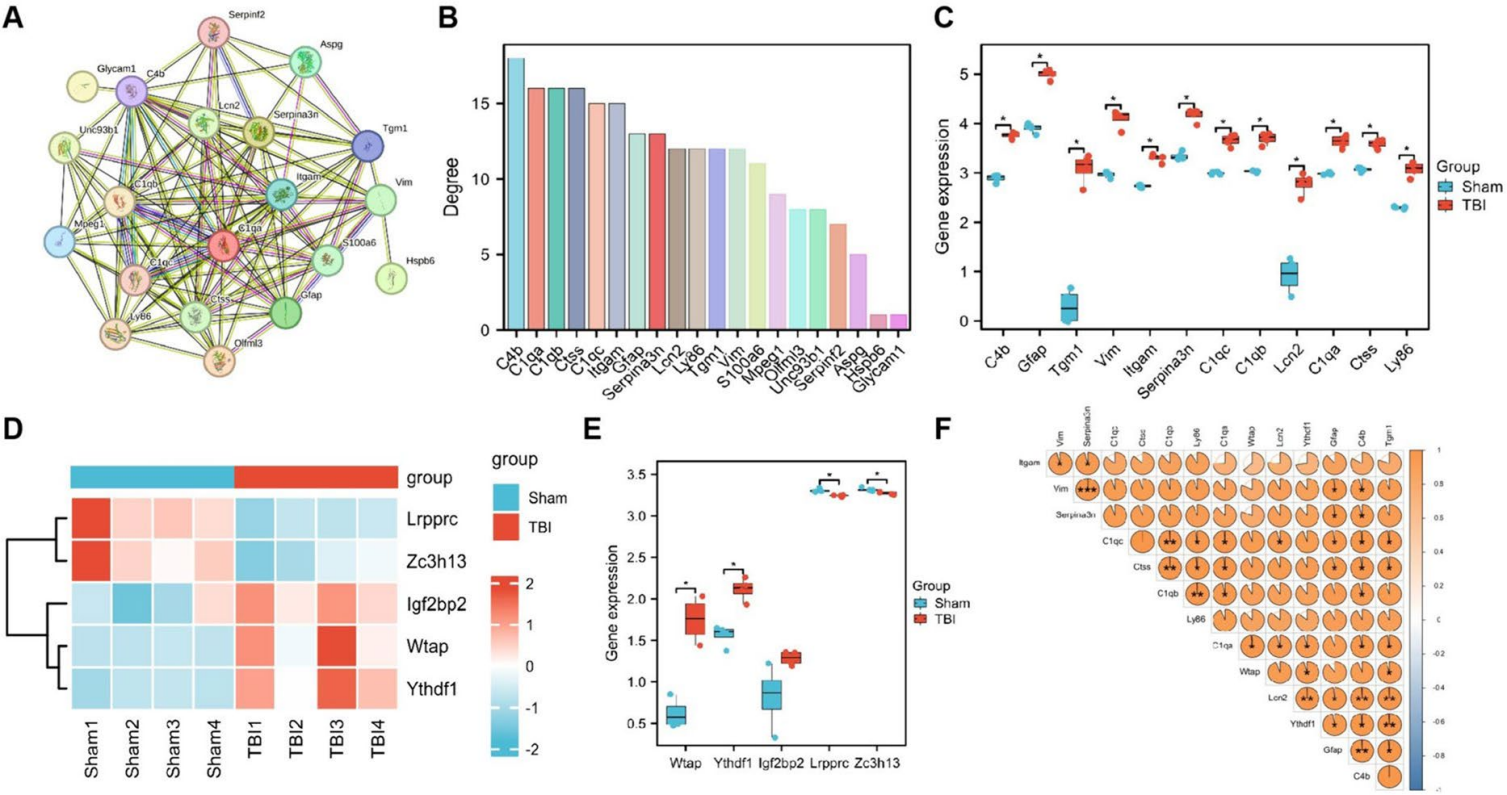
Selection of key target genes and m6A factors through PPI and correlation analysis. (**A**) PPI network of proteins encoded by the top 20 differential genes; (**B**) Bar graph showing the degree ranking of proteins encoded by the top 20 differential genes; (**C**) Box plot showing the differential expression of genes with high degree ranking; (**D**-**E**) Heatmap and box plot showing the differential expression of m6A-related factors; (**F**) Correlation analysis heatmap between m6A-related factors and candidate target genes. Sham group: *n* = 4, TBI group: *n* = 4, * indicates *P* < 0.05

In the subsequent step, a Protein–Protein Interaction (PPI) network analysis was performed on the proteins encoded by the top 20 genes exhibiting differences (Fig. 2A). The calculation of their degree centrality demonstrated that C4b, C1qa, C1qb, Ctss, C1qc, Itgam, Gfap, Serpina3n, Lcn2, Ly86, Tgm1, and Vim were ranked as having high degree centrality (Fig. 2B). Furthermore, an analysis of differential expression indicated that these 12 genes exhibited high expression in TBI mouse brain tissue, with Lcn2 and Tgm1 displaying the most pronounced changes in expression (Fig. 2C).

Modifying RNA with m6A (N6-methyladenosine) is the most prevalent among higher organisms. Research has demonstrated that m6A modification potentially ameliorates neuronal functional impairments caused by central nervous system (CNS) injuries such as TBI, cerebral hemorrhage, and ischemic stroke. Additionally, it can suppress cell apoptosis, inflammation, pyroptosis, and ferroptosis, making it a promising target for treating CNS injuries (Tian et al. 2022). However, a notable gap exists in the extensive exploration of the mechanisms of m6A in TBI. To gain deeper insights into the m6A-related mechanisms in TBI, we extracted the expression data of m6A-related factors from the transcriptome sequencing results. We identified two downregulated m6A factors (Lrpprc and Zc3h13) and three upregulated m6A factors (Wtap, Ythdf1, and Igf2bp2), with Wtap and Ythdf1 exhibiting the most differential expression (Fig. 2D-E). Further correlation analysis was performed on the highly ranked genes based on their degree and m6A factors Wtap and Ythdf1. The findings revealed a positive correlation between Wtap and Ythdf1. Additionally, Ythdf1 displayed a positive correlation with C1qa, Lcn2, Gfap, C4b, and Tgm1. Notably, the correlation between Ythdf1 and Lcn2, Tgm1, was the most (Fig. 2F).

Earlier investigations have reported that Lcn2 is upregulated in TBI and is crucial in regulating neuroinflammation associated with TBI. Lcn2 is subject to m6A modification, whereas the regulatory function of Tgm1 in TBI and its potential for m6A modification are yet to be determined (Kim et al. 2023; Tan et al. 2023). Therefore, we chose Lcn2 for further investigation. We further examined the levels of Wtap, Ythdf1, and Lcn2 in the cerebral cortex applying Western blot analysis. The results revealed an upregulation of Wtap, Ythdf1, and Lcn2 levels in the cerebral cortex of mice in the TBI group (Figure S2A). The quantification of m6A RNA methylation and dot plot analysis showed an elevation in the m6A modification level in the cerebral cortex of mice in the TBI group (Figure S2B-C). RT-qPCR and MeRIP qPCR assays illustrated that the levels of Lcn2 mRNA and m6A modifications in the cerebral cortex of TBI mice were elevated (Figure S2D-E).

Drawing from the aforementioned outcomes, a hypothesis emerges proposing that the mediation of Lcn2 m6A modification by Wtap/Ythdf1 may have a regulatory function in the advancement of TBI.

### Elucidating the regulation of Lcn2 through m6A modification mediated by Wtap and Ythdf1 in a primary cortical neuron model of TBI

The m6A methyltransferase, Wtap, is a crucial component of the m6A modification complex. Ythdf1, one of the m6A reader proteins, can identify m6A methylated mRNA and enhance mRNA stability and translation (You et al. 2022; Shen et al. 2021). Research indicates that WTAP could induce m6A modification on FOXO3a mRNA via m6A reader, thereby increasing the stability of FOXO3a mRNA (Wang et al. 2023a). Nonetheless, there is presently a lack of research documenting the regulatory role of Wtap/Ythdf1 in the m6A modification of Lcn2 in TBI.

**Fig. 3:**
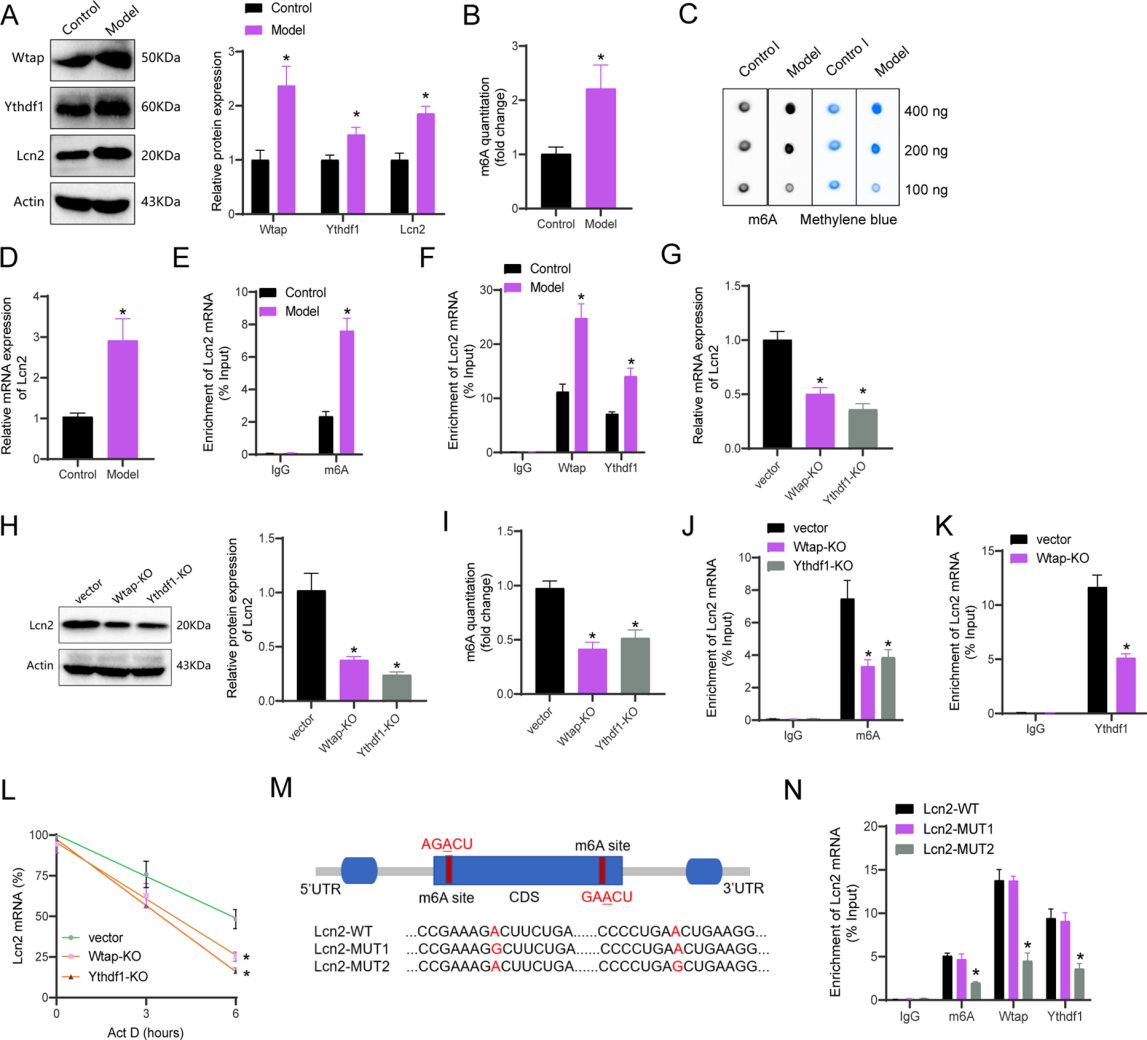
Wtap/Ythdf1 Mediates Lcn2 m6A Modification. (**A**) Western blot analysis of protein expression of Wtap, Ythdf1, and Lcn2 in primary cortical neurons of different groups; (**B**-**C**) Quantification of m6A RNA methylation and m6A dot plot experiments to detect m6A modification levels in primary cortical neurons of different groups; (**D**) RT-qPCR analysis of mRNA expression of Lcn2 in primary cortical neurons of different groups; (**E**) MeRIP qPCR analysis of m6A modification levels of Lcn2 in primary cortical neurons of different groups; (**F**) RIP PCR analysis of the enrichment of Wtap and Ythdf1 on Lcn2 mRNA; (**G**-**H**) RT-qPCR and Western blot analysis of Lcn2 expression in primary cortical neurons after Wtap or Ythdf1 knockout; (**I**) Quantification of m6A RNA methylation and m6A dot plot experiments to detect changes in m6A modification levels after Wtap or Ythdf1 knockout; (**J**) MeRIP qPCR analysis of m6A modification levels of Lcn2 after Wtap or Ythdf1 knockout; (**K**) RIP PCR analysis of the enrichment of Ythdf1 on Lcn2 mRNA after Wtap knockout; (**L**) RT-qPCR analysis of the mRNA half-life of Lcn2 after Wtap or Ythdf1 knockout, Act D (actinomycin D, 5 µg/mL); (**M**) Schematic representation of the location and mutation of m6A motifs in the coding sequence (CDS) of Lcn2 mRNA; (**N**) RIP PCR analysis of the binding of m6A, Wtap, and Ythdf1 to Lcn2 mRNA before and after Lcn2 mutation. * indicates *P* < 0.05 compared to the Control, vector, or Lcn2-WT group; cell experiments repeated 3 times

For this purpose, primary cortical neurons were cultured and subsequently identified using immunofluorescent staining. The immunofluorescent staining revealed that the cell bodies and neurites of the neurons exhibited a red stain with β-III tubulin. These results indicate that around 90% of the cells were identified as neurons (Figure S3A). An *in vitro* TBI model was developed to investigate release of extracellular LDH and neuronal cell death. The results showed that LDH release and neuronal apoptosis increased in the Model group relative to the Control group (Figure S3B-C). Western blot analysis displayed enhanced expression levels of Wtap, Ythdf1, and Lcn2 in the Model group compared to the Control group (Fig. 3A). Quantification of m6A RNA methylation and Dot plot analysis demonstrated an escalation in the levels of m6A modification in primary cortical neurons of the Model group (Fig. 3B-C). RT-qPCR and MeRIP qPCR analyses demonstrated increases in the mRNA and m6A modification levels of Lcn2 in the Model group in comparison to the other groups (Fig. 3D-E). Further observations from RIP-PCR experiments revealed that the Model group exhibited binding of Wtap and Ythdf1 with Lcn2 mRNA when contrasted with the Control group (Fig. 3F).

Consequently, the CRISPR/Cas9 gene editing system was employed to disrupt the Wtap and Ythdf1 genes in primary cortical neurons, as illustrated in the plasmid construction presented in Figure S4A. The successful deletion of the targeted sequences was verified using Sanger sequencing and PCR analysis (Figure S4B-C). Analysis through Western blot demonstrated that the detection of Wtap or Ythdf1 in the KO group was markedly lower relative to the wild type (WT) (Figure S4D). The expression of Lcn2 decreased after knockout of Wtap or Ythdf1, as confirmed by RT-qPCR and Western blot analysis (Fig. 3G-H). Subsequently, we examined the alterations in m6A modification levels in TBI model cells following the knockout of the Wtap and Ythdf1 genes. The findings demonstrated a decrease in the cells' total m6A modification level and the Lcn2 m6A modification level after the knockout of either Wtap or Ythdf1 (Fig. 3I-J). RIP-PCR experiments indicated a decline in the binding of Ythdf1 to Lcn2 mRNA upon the knockout of Wtap (Fig. 3K). The RNA decay rate assay results indicated that the deletion of Wtap or Ythdf1 decreased the half-life of Lcn2 mRNA (Fig. 3L).

Finally, the m6A binding sites of Lcn2 were predicted using the SRAMP website. Multiple m6A binding sites were identified on the RNA sequence of the Lcn2 gene (Figure S5A). Figure S5B depicts two high-confidence m6A sites. Mutations were incorporated into the target site to create wild-type (Lcn2-WT) and mutant (Lcn2-MUT1 and Lcn2-MUT2) vectors (Fig. 3M). The RIP PCR results revealed that Lcn2-MUT2 effectively hindered the interaction between m6A, Wtap, Ythdf1, and Lcn2 mRNA (Fig. 3N).

These results indicate that the knockout of Wtap and Ythdf1 could suppress Lcn2 m6A modification in primary cortical neurons of the TBI model.

### Understanding the impact of Wtap/Ythdf1 knockdown on Lcn2 expression, neuronal death, and inflammation in a TBI model and the reversal Effects of Lcn2 overexpression

**Fig. 4:**
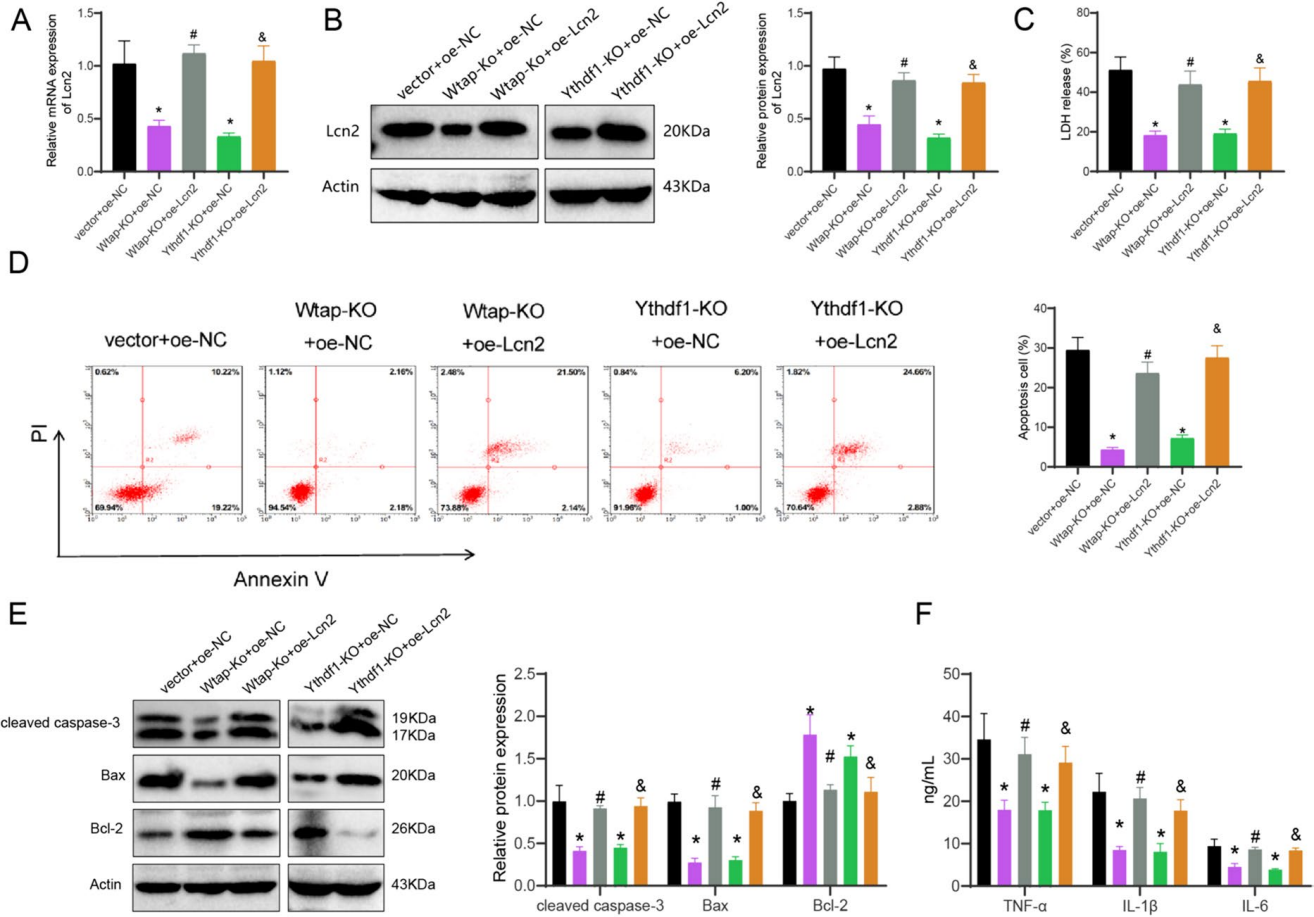
Regulation of Lcn2 Expression by Wtap/Ythdf1 Affects Neuronal Death in TBI Model. (**A**-**B**) RT-qPCR and Western blot analysis of Lcn2 expression in primary cortical neurons of different groups; (**C**) Detection of LDH release in primary cortical neurons of different groups; (**D**) Flow cytometry analysis of apoptosis in primary cortical neurons of different groups; (**E**) Western blot analysis of the expression of apoptosis-related proteins in primary cortical neurons of different groups; (**F**) ELISA analysis of the levels of inflammatory factors TNF-α, IL-1β, and IL-6 in primary cortical neurons of different groups. * indicates P < 0.05 compared to the vector + oe-NC group, # indicates *P* < 0.05 compared to the Wtap-KO + oe-NC group, & indicates *P* < 0.05 compared to the Ythdf1-KO + oe-NC group; cell experiments repeated 3 times

In the following analysis, we explore the potential role of Wtap/Ythdf1 in regulating Lcn2 expression and its impact on neuronal death in the TBI model. Lcn2 was overexpressed in primary cortical neurons of a TBI model with knockdown of Wtap/Ythdf1. RT-qPCR and Western blot analysis demonstrated a minimization in Lcn2 expression following the knockdown of Wtap/Ythdf1. However, the downregulation of Lcn2 caused by knockdown of Wtap/Ythdf1 was reversed upon overexpression of Lcn2 (Fig. 4A-B). The detection of extracellular LDH release and neuronal apoptosis showed that the knockdown of Wtap/Ythdf1 decreased both LDH release from cells and neuronal apoptosis, whereas the overexpression of Lcn2 increased both LDH release from cells and neuronal apoptosis (Fig. 4C-D). The Western blot analysis was carried out to investigate the expression levels of proteins associated with apoptosis. The findings indicated a drop in the manifestation of cleaved caspase-3 and Bax proteins upon Wtap/Ythdf1 knockout, while an elevation was noted in the expression of the anti-apoptotic factor, Bcl-2 protein. Conversely, overexpressing Lcn2 increased cleaved caspase-3 and Bax proteins and decreased expression of the anti-apoptotic factor, Bcl-2 protein (Fig. 4E). ELISA detection revealed a cutback in inflammatory factors TNF-α, IL-1β, and IL-6 levels following Wtap/Ythdf1 knockout. Conversely, overexpression of Lcn2 increased TNF-α, IL-1β, and IL-6 (Fig. 4F).

The results above indicate that the knockout of Wtap/Ythdf1 could downregulate the expression of Lcn2, reducing neuronal death and inflammatory reactions. Overexpressing Lcn2 could reverse the effects of the Wtap/Ythdf1 knockout.

### Exploration of microglial and astrocyte activation and the regulatory role of Lcn2 in TBI

**Fig. 5:**
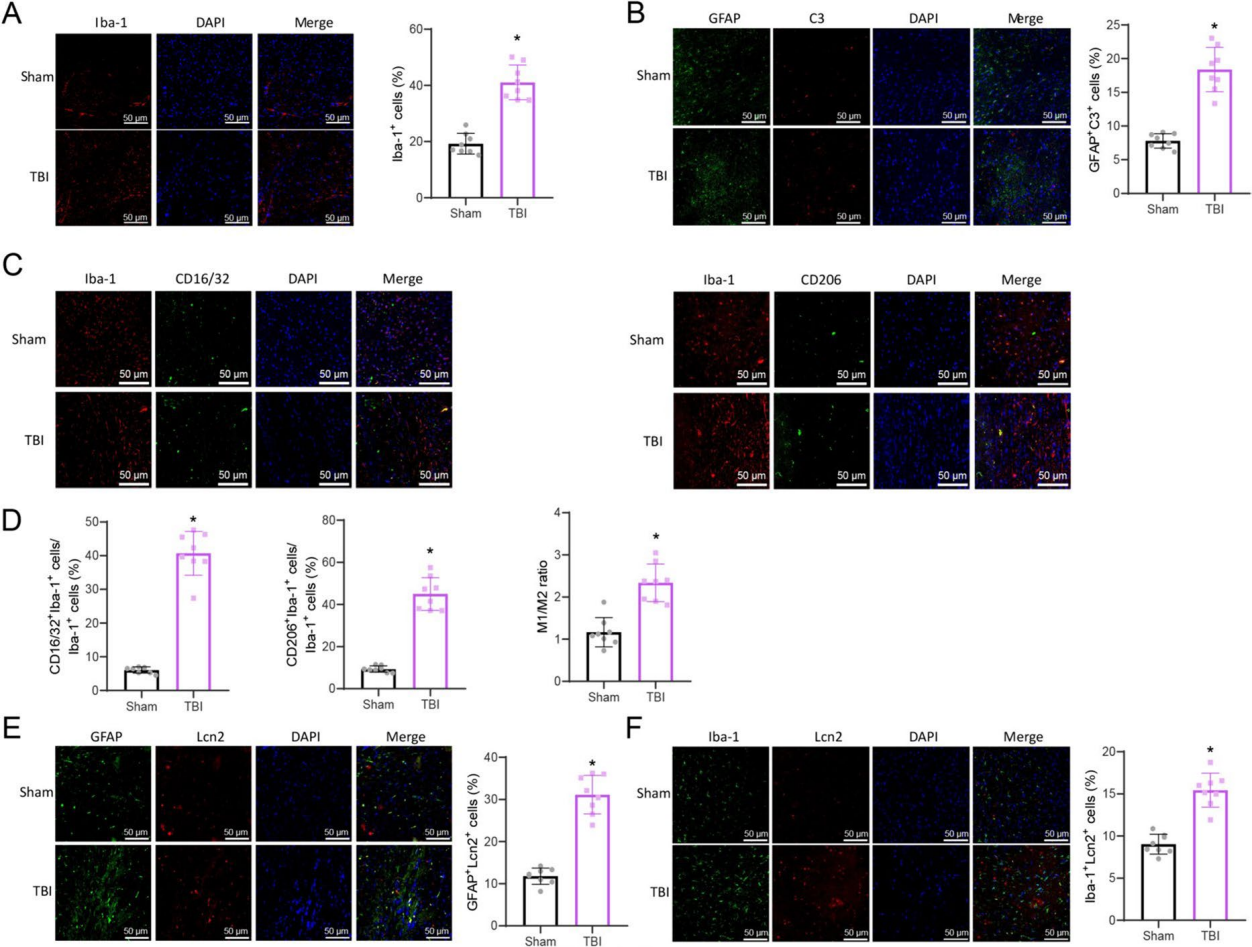
Activation of Reactive Astrocytes and Microglia, and Expression of Lcn2 in TBI. (**A**-**B**) Immunofluorescence staining of Iba-1-positive and GFAP/C3 double-positive cells in the cortex of Sham and TBI mice (scale bar = 50 μm); (**C**-**D**) Immunofluorescence staining of M1-like and M2-like microglia in the cortex of different groups of mice (scale bar = 50 μm); (**E**–**F**) Immunofluorescence staining of Lcn2 and GFAP double-positive, as well as Lcn2 and Iba-1 double-positive cells in the cortex of different groups of mice (scale bar = 50 μm). * indicates *P* < 0.05 compared to the Sham group; each group consisted of 8 mice

Microglia and astrocytes regulate the neuroimmune response after TBI (Todd et al. 2021; Karve et al. 2016). Initially, the activated status of microglia and astrocytes in the cortical area of TBI mouse models was evaluated. The immunofluorescence staining results demonstrated a boost in the percentage of Iba-1 positive cells and GFAP/C3 double-positive cells in the cerebral cortex of mice in the TBI group in juxtaposition with the Sham group. This increase indicates the activation of microglial cells and reactive astrocytes (Fig. 5A-B). Additionally, we conducted double-staining of microglia using the Iba-1 marker, the M1-related marker CD16/32, and the M2-related marker CD206. Our findings revealed an increase in the ratio of CD16/32 and Iba-1 double-positive M1-like cells in the cerebral cortex of mice in the TBI group. Likewise, there was an increase in the proportion of CD206 and Iba-1 double-positive M2-like cells. Furthermore, the ratio of M1/M2 cells was also increased (Fig. 5C-D). The results are consistent with the literature reports (Chen et al. 2018; Wu et al. 2021a). TBI triggers microglial activation and their transformation into a pro-inflammatory M1-like phenotype.

Academic investigations have demonstrated that Lcn2 can regulate the activation of neurotoxic microglia and astrocytes (Wang et al. 2021b). Differential analysis was performed on the public database sequencing dataset GSE167459 to obtain volcano plots and heat maps of differentially expressed genes in microglial cells and astrocytes (Figure S6A-B). The analysis results revealed the upregulation of Lcn2 in astrocytes in TBI mice. In microglial cells, Lcn2 expression showed an upward trend in the TBI group in contrast to the Sham group; however, the variance did not reach statistical significance (Figure S6C).

Further immunofluorescence staining results indicate that in the mouse cerebral cortex of the TBI group, there is a significant growth in the ratio of cells that are double-positive for Lcn2 and NeuN, as well as for Lcn2 and GFAP. Additionally, there is a noticeable elevation in the percentage of cells that are double-positive for Lcn2 and Iba-1 (Figure S7A; Fig. 5E-F).

The results above indicate that reactive astrocytes and microglia are activated in TBI, and Lcn2 may regulate their activation.

### Neuron-Derived Lcn2 promotes activation of reactive astrocytes and M1-Like inflammatory microglia in TBI

Multiple studies have reported that neurons can release Lcn2 to activate glial cells (Xing et al. 2014). To scrutinize the potential involvement of Lcn2 in mediating the interaction between neurons and glial cells (astrocytes and microglia) in TBI, we isolated and cultured primary astrocytes and microglia from mice. Immunofluorescence and flow cytometry were employed to characterize the isolated cells. The results demonstrated that more than 95% of the cells were positive for Iba-1 or CD11b in our primary microglia culture. Additionally, in the primary astrocyte culture, over 95% of the cells showed GFAP positivity (Figure S7B-D).

**Fig. 6:**
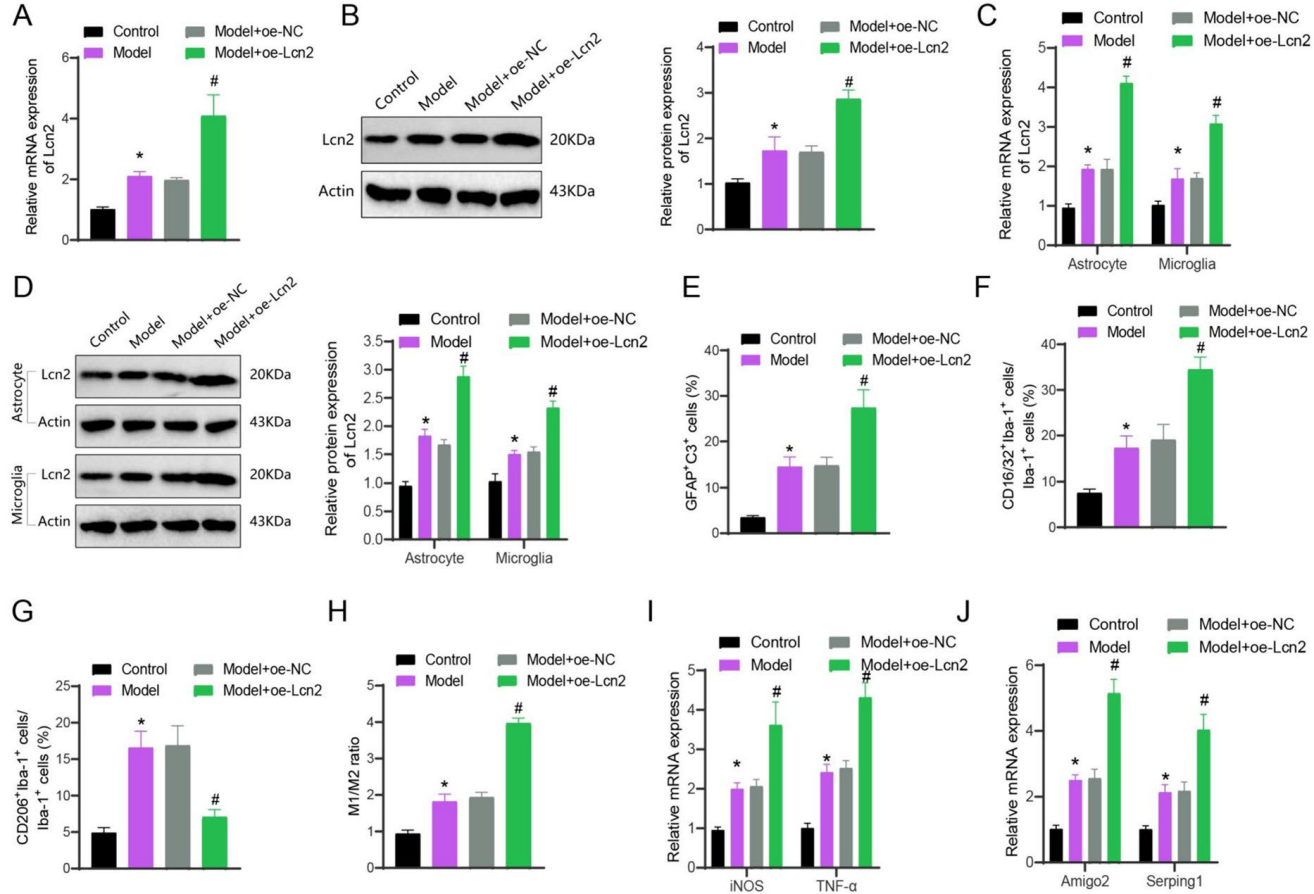
Regulation of neural glial cell activation by Lcn2 secreted by cortical neurons. (**A**-**B**) Expression of Lcn2 in primary cortical neurons from each group detected by RT-qPCR and Western blot; (**C**-**D**) Expression of Lcn2 in small glial cells and astrocytes from primary cortical neurons treated with different conditioned media, detected by RT-qPCR and Western blot; (**E**) Proportion of GFAP/C3 double-positive cells in astrocytes from each group; (**F**) Proportion of M1-like cells in each group of microglia; (**G**) Proportion of M2-like cells in each group of microglia; (**H**) Proportion of M1-like and M2-like small glial cells from each group; (**I**) Expression of iNOS and TNF-α in small glial cells from each group detected by RT-qPCR (**J**) Expression of Amigo2 and Serping1 in astrocytes from each group detected by RT-qPCR. * indicates difference from Control group, *p* < 0.05, # indicates difference from Model + oe-NC group, *p* < 0.05, the experiment was repeated 3 times

Additionally, we conducted overexpression of Lcn2 in primary cortical neurons from individuals with TBI. The expression of Lcn2 was assessed through both RT-qPCR and Western blot. As opposed to the Control or Model + oe-NC group, the expression of Lcn2 increased in the Model and Model + oe-Lcn2 groups (Fig. 6A-B). The conditioned medium from each distinct group of primary cortical neurons was introduced to the astrocytes, and the expression of Lcn2 was measured. The findings indicated an upsurge in the presence of Lcn2 in the astrocytes and microglia of both the Model and Model + oe-Lcn2 groups, compared to the Control or Model + oe-NC group. (Fig. 6C-D).

Further immunofluorescence staining revealed a growth in the ratio of GFAP/C3 double-positive cells in astrocytes in the Model group compared to the Control group. Additionally, the M1-like and M2-like cells in microglia increased in the Model group, increasing the M1/M2 ratio. Similarly, compared to the Model + oe-NC group, the Model + oe-Lcn2 group displayed an escalation in GFAP/C3 double-positive cells in astrocytes. Moreover, the Model + oe-Lcn2 group showed an elevation in the proportion of M1-like cells in microglia, along with a downtick in the percentage of M2-like cells, leading to a substantial increase in the M1/M2 ratio (Fig. 6E-H; Figure S8A-C). Furthermore, we observed the presence of pro-inflammatory factors in microglia and reactive astrocyte-specific transcription factors. The research illustrated an augmentation in iNOS and TNF-α expression levels in microglia in both the Model and Model + oe-Lcn2 groups, relative to the Control or Model + oe-NC group. Additionally, we observed a rise in the Amigo2 expression levels of Amigo2 and Serping1 in reactive astrocytes (Fig. 6I-J).

The results above demonstrate an upregulation of Lcn2 in cortical neurons, which induces the activation of reactive astrocytes and M1-like inflammatory microglia.

### Induction of apoptosis in cortical neurons by M1-Like microglial cells and reactive astrocytes

**Fig. 7:**
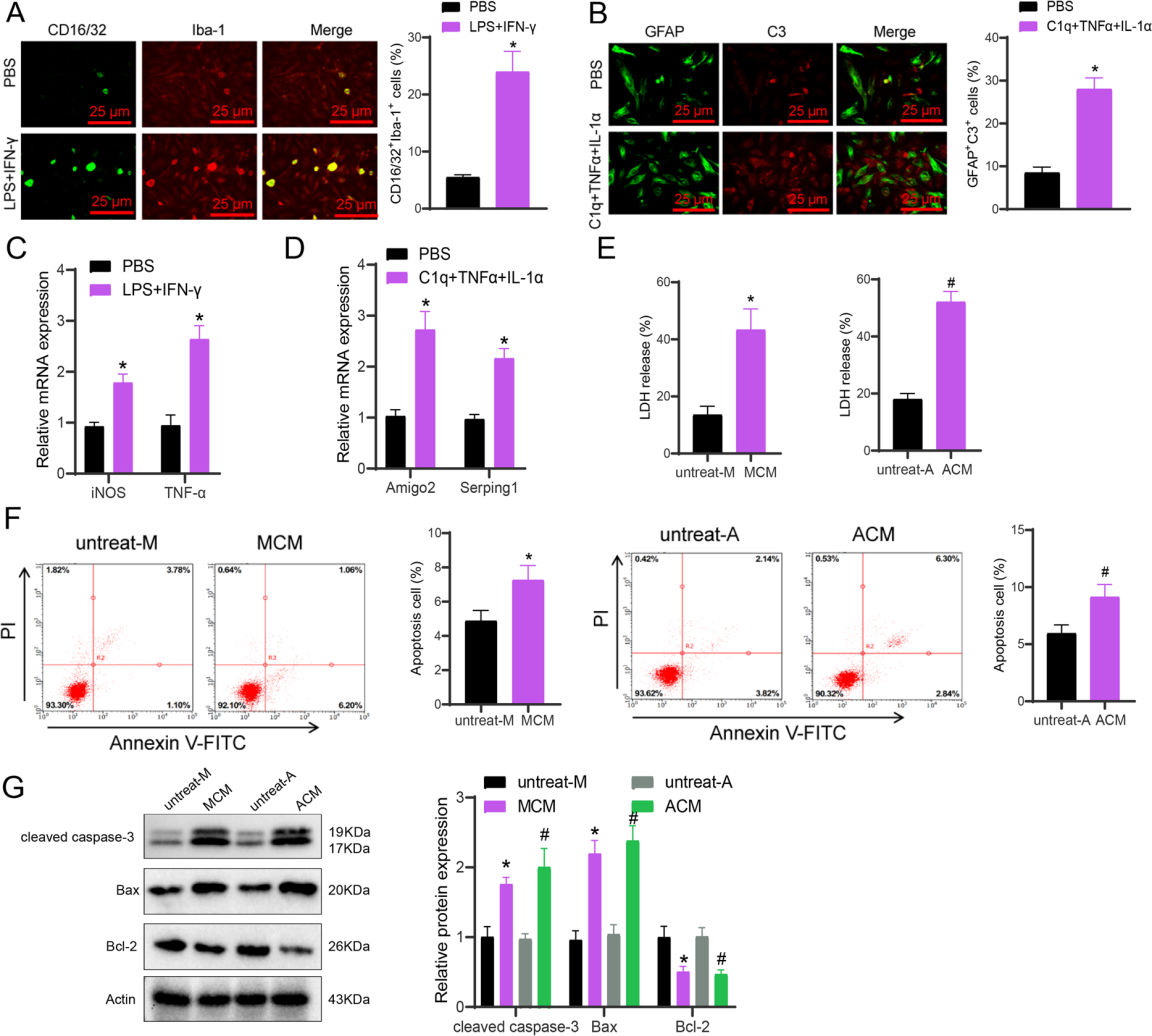
Regulation of cortical neuron apoptosis by activated glial cells. (**A**) Proportion of M1-like glial cells in small glial cells from each group detected by immunofluorescence staining (scale bar = 50 μm); (**B**) Proportion of GFAP/C3 double-positive cells in astrocytes from each group detected by immunofluorescence staining (scale bar = 50 μm); (**C**) Expression of iNOS and TNF-α in small glial cells from each group detected by RT-qPCR; (**D**) Expression of Amigo2 and Serping1 in astrocytes from each group detected by RT-qPCR; (**E**) LDH release in primary cortical neurons from each group treated with MCM or ACM; (**F**) Apoptosis in primary cortical neurons from each group treated with MCM or ACM detected by flow cytometry; (**G**) Expression of apoptosis-related proteins in primary cortical neurons from each group treated with MCM or ACM detected by Western blot. MCM: M1-like glial cell conditioned medium; ACM: reactive astrocyte conditioned medium; untreat-M and untreat-A are small glial cell and astrocyte culture media without induction; * indicates difference from PBS or untreat-M group, p < 0.05, # indicates difference from untreat-A group, *p* < 0.05, the experiment was repeated 3 times

Moreover, we are further examining the toxic effects of reactive astrocytes and the activation of M1-like inflammatory microglia on neurons. Microglia could be activated using LPS and IFN-γ, while reactive astrocytes could be activated using C1q, TNF-α, and IL-1α. The immunofluorescence analysis revealed an augmentation in the total of M1-like microglia after LPS and IFN-γ induction. Similarly, reactive astrocytes increased following C1q, TNF-α, and IL-1α induction (Fig. 7A-B). The results from RT-qPCR detection demonstrated an upturn in the expression of iNOS and TNF-α in microglial cells after induction with LPS and IFN-γ. Besides, induction with C1q, TNF-α, and IL-1α (Fig. 7C-D) increased the expression of Amigo2 and Serping1 in reactive astrocytes.

Further investigation involved adding microglia-conditioned medium (MCM) and astrocyte-conditioned medium (ACM) to primary cortical neurons. We examined LDH release and cell apoptosis and found that both apoptosis and LDH release increased in the MCM and ACM groups compared to the untreated-M or untreated-A groups (Fig. 7E-F). Investigation of apoptosis-related protein expression was carried out through Western blot analysis. Observations indicated that cleaved caspase-3 and Bax protein expression showed a rise in the MCM and ACM cohorts as opposed to the untreat-M or untreat-A groups. Conversely, the anti-apoptotic factor Bcl-2 protein's expression decreased (Fig. 7G).

The results above revealed that M1-like microglial cells and reactive astrocytes are capable of inducing apoptosis in cortical neurons.

### Inhibition of Wtap reduces Lcn2 expression and mitigates neuroinflammation and neuronal damage following TBI

**Fig. 8:**
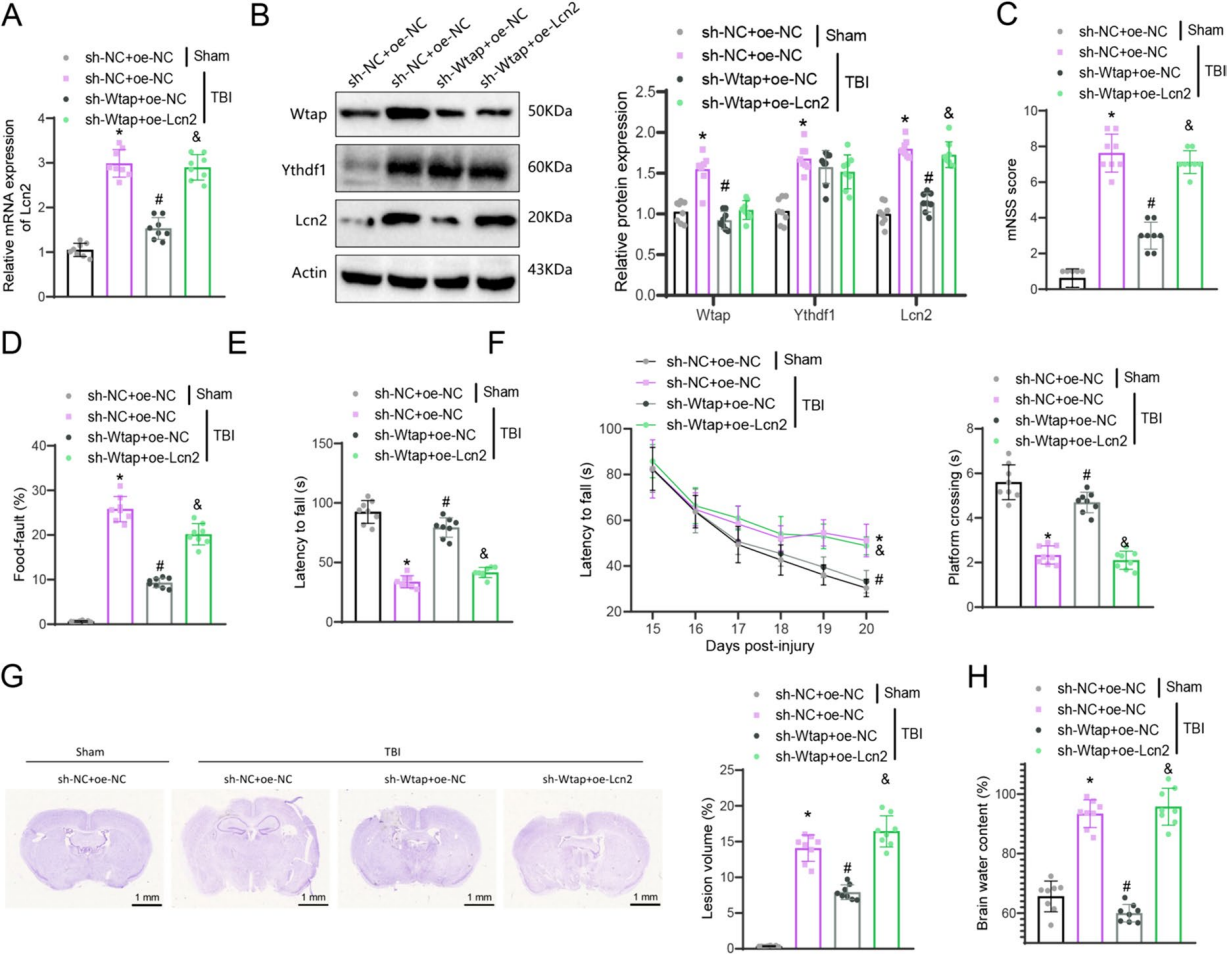
Regulation of brain tissue and neurofunctional disorders in TBI mice by the Wtap/Lcn2 axis. (**A**-**B**) Expression of Wtap, Ythdf1, and Lcn2 in the cortical tissues of mice from each group detected by RT-qPCR and Western blot; (C-E) mNSS scores (**C**), foot fault rates (**D**), and latency to fall in the rotarod test (**E**) of mice from each group; (**F**) Escape latency and time to cross the platform in the MWM test of mice from each group; (**G**) Contusion volume in the ipsilateral cortex of mice from each group (scale bar = 1 mm); (**H**) Water content in the brain tissues of mice from each group. * indicates difference from Sham + sh-NC + oe-NC group, *p* < 0.05, # indicates difference from TBI + sh-NC + oe-NC group, p < 0.05, & indicates difference from TBI + sh-Wtap + oe-NC group, *p* < 0.05, each group consisted of 8 mice

Finally, we conducted additional *in vivo* animal experiments to further validate how Wtap/Ythdf1 regulates Lcn2 expression and impacts secondary neural damage following TBI. The drop in Wtap expression and the growth in Lcn2 expression were achieved through stereotactic injection of a low-dose Wtap and overexpression of Lcn2 lentivirus into the brains of TBI mice. Firstly, we gauged the expression of relevant factors in the cerebral cortex tissue of each mouse group. The results suggested that when contrasted with the Sham + sh-NC + oe-NC group, the protein expression of Wtap and Ythdf1 in the TBI + sh-NC + oe-NC group increased, and both the mRNA and protein expressions of Lcn2 also increased. In contrast with the TBI + sh-NC + oe-NC group, the expression of Wtap and Lcn2 decreased in the TBI + sh-Wtap + oe-NC group, whereas the protein expression of Ythdf1 manifested no adjustment. Additionally, in relation to the TBI + sh-Wtap + oe-NC group, the expression of Lcn2 increased in the TBI + sh-Wtap + oe-Lcn2 group, while the protein expression of Wtap and Ythdf1 showed no change (Fig. 8A-B).

The mice in each group underwent neurological behavioral tests and evaluations. The TBI + sh-NC + oe-NC group exhibited elevated mNSS score, impaired foot movement, and decreased latency in the rotating rod test compared to the Sham + sh-NC + oe-NC group. In contrast, the TBI + sh-Wtap + oe-NC group displayed a decrease in mNSS score and impaired foot movement, along with an increase in latency when juxtaposed with the TBI + sh-NC + oe-NC experimental group. Moreover, the TBI + sh-Wtap + oe-Lcn2 group exhibited an increase in mNSS score and impaired foot movement, as well as a decrease in latency, contrasting with the TBI + sh-Wtap + oe-NC group (Fig. 8C-E). MWM experiment outcomes demonstrated that the escape latency of mice in the TBI + sh-NC + oe-NC group was prolonged compared to the Sham + sh-NC + oe-NC group.

Additionally, the time to cross the platform was reduced. Moreover, when compared to the TBI + sh-NC + oe-NC group, the TBI + sh-Wtap + oe-NC group exhibited a shortened escape latency and prolonged time to cross the platform. Furthermore, the TBI + sh-Wtap + oe-Lcn2 group indicates prolonged escape latency and reduced time to cross the platform (Fig. 8F). Further investigation revealed that the TBI + sh-NC + oe-NC group exhibited brain tissue damage and edema in comparison with the Sham + sh-NC + oe-NC group. In contrast, the TBI + sh-Wtap + oe-NC group demonstrated a notable reduction in brain tissue damage and edema compared to the TBI + sh-NC + oe-NC group. Furthermore, the TBI + sh-Wtap + oe-Lcn2 group displayed an exacerbation of brain tissue damage and edema compared to the TBI + sh-Wtap + oe-NC group in mice (Fig. 8G-H).

**Fig. 9:**
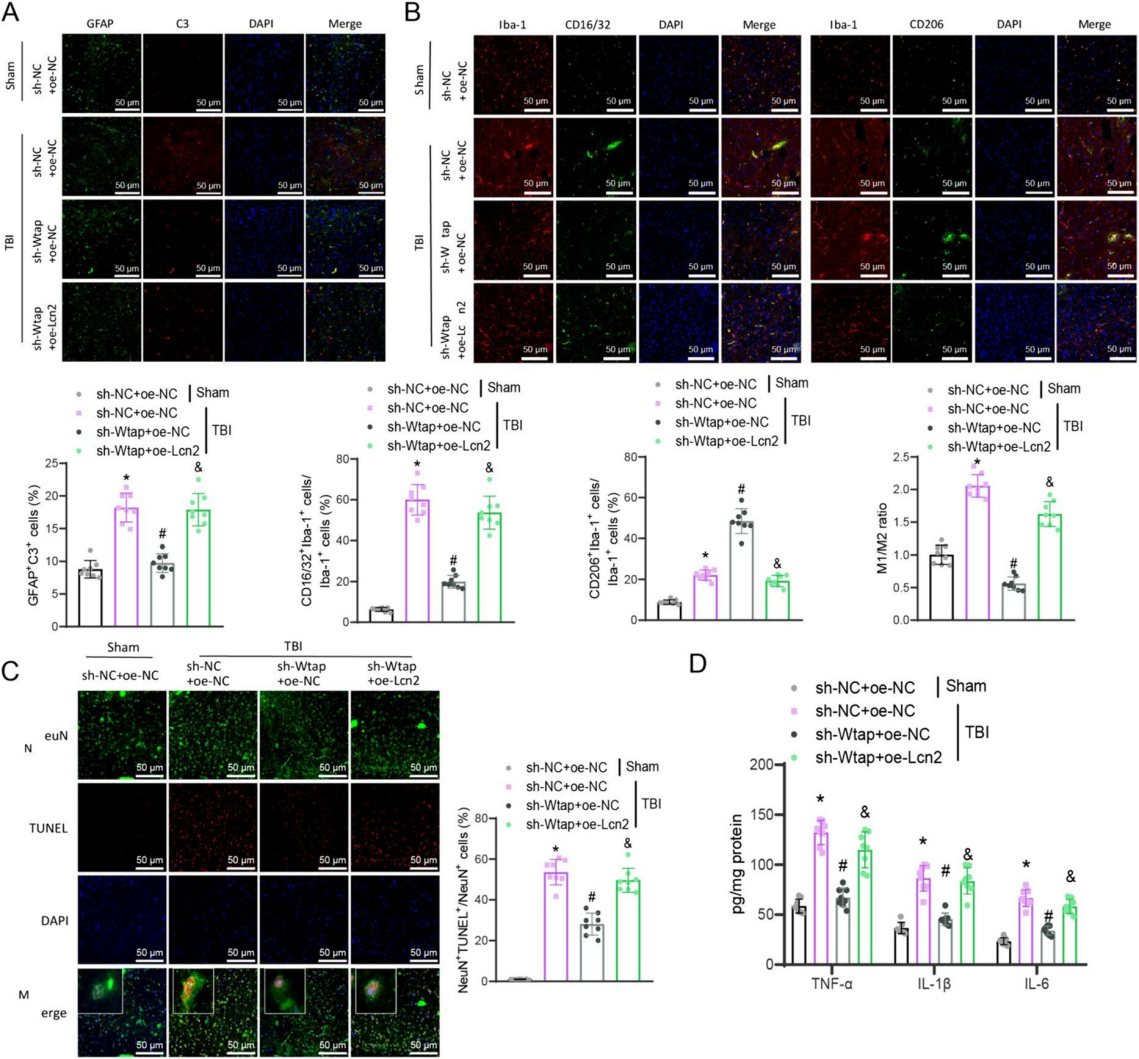
Regulation of cortical neuron apoptosis and glial cell activation in TBI mice by the Wtap/Lcn2 axis. (**A**) Proportion of GFAP/C3 double-positive cells in the cortical tissues of mice from each group detected by immunofluorescence staining (scale bar = 50 μm); (**B**) Proportion of M1-like and M2-like glial cells in the cortical tissues of mice from each group detected by immunofluorescence staining (scale bar = 50 μm); (**C**) Apoptosis of neurons in the cortical tissues of mice from each group detected by TUNEL staining (scale bar = 200 μm); (**D**) Expression of pro-inflammatory factors in the cortical tissues of mice from each group detected by ELISA. * indicates difference from Sham + sh-NC + oe-NC group, *p* < 0.05, # indicates difference from TBI + sh-NC + oe-NC group, *p* < 0.05, & indicates difference from TBI + sh-Wtap + oe-NC group, *p* < 0.05, each group consisted of 8 mice

Further investigation into the activation of glial cells in brain tissue revealed that the TBI + sh-NC + oe-NC group had higher proportions of reactive astrocytes, M1-like microglia, and M2-like microglia, along with an increased M1/M2 ratio compared to the Sham + sh-NC + oe-NC group. Conversely, the TBI + sh-Wtap + oe-NC group exhibited lower proportions of reactive astrocytes and M1-like microglia while showing increased proportions of M2-like microglia, resulting in a decreased M1/M2 ratio relative to the TBI + sh-NC + oe-NC group. Additionally, the TBI + sh-Wtap + oe-Lcn2 group showed higher proportions of reactive astrocytes and M1-like microglia, along with decreased proportions of M2-like microglia, leading to an increased M1/M2 ratio when contrasted with the TBI + sh-Wtap + oe-NC group (Fig. 9A-B).

Finally, we also investigated the extent of neuronal apoptosis and inflammatory markers in brain tissues. The TBI + sh-NC + oe-NC group revealed escalated neuronal apoptosis and pro-inflammatory factors in relation to the Sham + sh-NC + oe-NC group. On the other hand, the TBI + sh-Wtap + oe-NC group showed a notable decrease in neuronal apoptosis and pro-inflammatory factors in contrast with the TBI + sh-NC + oe-NC group. Additionally, there was a remarkable increase in neuronal apoptosis and pro-inflammatory factors in the TBI + sh-Wtap + oe-Lcn2 group in comparison with the TBI + sh-Wtap + oe-NC group (Fig. 9C-D).

The above results suggest that the inhibition of Wtap could reduce the expression of Lcn2, suppress the activation of reactive astrocytes and pro-inflammatory M1-like microglial cells, as well as improve neuroinflammation, neuronal apoptosis and functional impairments in mice in the aftermath of TBI.

## Discussion

This study validates the fundamental functions of Wtap and Ythdf1 in the m6A modification of Lcn2. Preceding research has demonstrated the engagement of Wtap and Ythdf1 in various physiological and pathological processes associated with m6A modification. However, their roles in TBI have not been elucidated yet (Jiang et al. 2021; Wang et al. 2020). This study is the first to demonstrate that Wtap and Ythdf1 could mediate m6A modification of Lcn2, thereby enhancing our understanding of the roles of these two factors in neurotoxicity. Previous studies have demonstrated the diverse role that glial cells play in neural injury and repair (Liu et al. 2021). This study supplies extra confirmation that the overexpression of Lcn2 could induce the activation of neurotoxic glial cells, aligning with the pro-inflammatory role of glial cells observed in prior research (Park et al. 2021).

Lipocalin-2 (Lcn2) is a protein synthesized and secreted by microglia, astrocytes, neurons, and endothelial cells in response to stress, such as inflammation and trauma (Jha et al. 2015). Lcn2 is implicated in the pathophysiology of different neurological disorders. Previous studies have shown that chronic exposure of the brain to Lcn2 can lead to neurofunctional dysregulation and cognitive impairment, while reducing Lcn2 expression can alleviate neuroinflammatory damage. However, the exact role of Lcn2 in TBI remains unclear (Appel et al. 2021; Olson et al. 2021; Jung et al. 2023). This study has elucidated the pivotal function of Lcn2 in TBI using experimental methods, which aligns with previous research findings emphasizing the involvement of Lcn2 in various neurological disorders. The m6A modification is considered an important mechanism that regulates gene expression (Mo et al. 2022). Prior research has demonstrated the involvement of m6A modification in numerous diseases. However, neurological injury's specific mechanism remains incompletely understood (Tian et al. 2022). This study enhances our comprehension of the function of m6A modification in neural injuries. It has been demonstrated that by regulating the activity or expression of Wtap and Ythdf1, a novel therapeutic target for TBI may be identified. This offers a new perspective for future TBI treatments, complementing other research in the field. Previous research has shown that Wtap-mediated m6A modification plays a vital protective function in the Purkinje cells of the brain (Yang et al. 2022). However, our experiments have revealed that Wtap and Ythdf1 can induce secondary damage post-TBI through mediating m6A modification of Lcn2, suggesting varying roles of Wtap in different cells within the nervous system, necessitating further exploration. Considering the roles of Wtap and Ythdf1 in Lcn2 m6A modification and the activation of neurotoxic glial cells in TBI, future studies could delve into the detailed interaction mechanisms between these factors and Lcn2. Additionally, investigating the potential roles of these factors in other crucial molecules in TBI, such as substance P, and their implications in other neurological disorders, will be valuable.

**Fig. 10:**
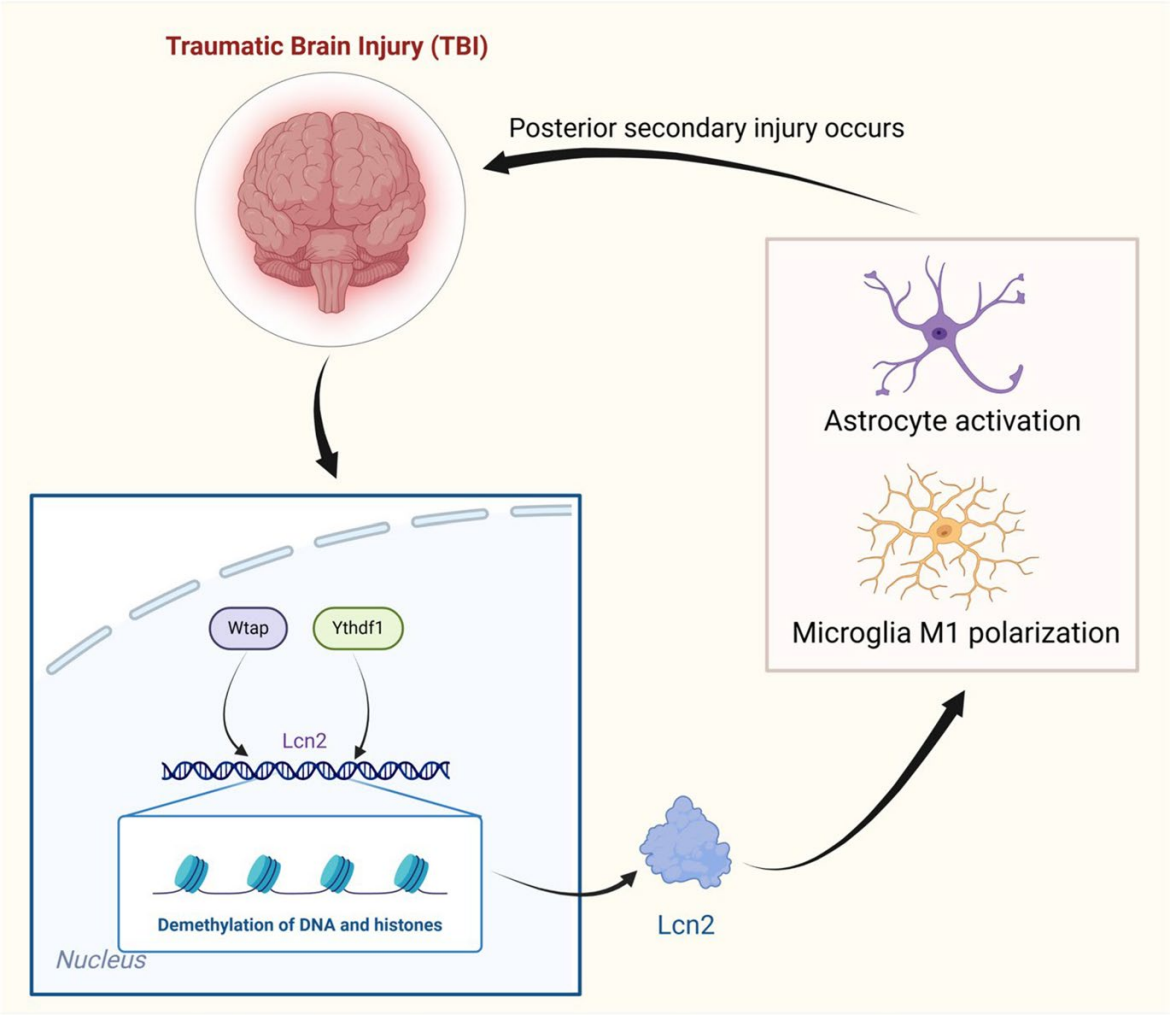
Schematic representation of the molecular mechanism by which the Wtap/Ythdf1-mediated neural Lcn2 m6A modification regulates the activation of astrocytes and microglia, affecting secondary damage in TBI mice

Based on the above findings, we could preliminarily derive the subsequent conclusions: Wtap and Ythdf1 promote the m6A modification of neuronal Lcn2. Neuronal secretion of Lcn2 further enhances reactive astrocyte activation and microglia M1 polarization, thereby inducing secondary injury in mice following TBI (Fig. 10). This study comprehensively examines the molecular mechanisms that drive subsequent damage after TBI, focusing on the crucial role of Wtap/Ythdf1-mediated Lcn2 m6A modification in TBI. It establishes a theoretical foundation for the field of neuroscience and proposes novel strategies for potential therapeutic interventions in clinical TBI. These findings may be advantageous for developing drugs targeting these particular molecular pathways.

Although mouse models provide valuable information, their physiological and biochemical characteristics differ from humans; therefore, these findings need further validation in more models. This study focuses on the roles of Wtap and Ythdf1, yet the m6A modification network involves numerous molecules and pathways, including the dynamic regulation among other m6A methyltransferases, reader proteins, and eraser proteins (Zaccara et al. 2019; Boulias and Greer 2023). Hence, further research is necessary to reveal a more comprehensive regulatory network. Additionally, this study defines the functional states of microglial cells using M1/M2 polarization; however, recent studies have shown that this definition is imprecise as microglial cells exhibit a diverse array of polarization programs. Future research should consider the various possible subtypes of microglial cells and conduct a comprehensive analysis (Wang et al. 2023c; Ransohoff 2016). This study focuses primarily on the short-term and medium-term effects post-TBI, yet additional investigation is required to explore the lasting effects of TBI and its relationship with m6A modification. To enhance understanding of the roles of Wtap and Ythdf1 in TBI, future studies could explore these findings in more animal models, cell models, and even human samples. Further exploration of the interactions between Wtap, Ythdf1, and other m6A-related molecules, as well as their roles in other neurological disorders, will contribute to a more comprehensive comprehension of the biological significance of m6A modification. Building upon the discoveries of this study, the identification or development of drugs targeting Wtap, Ythdf1, or Lcn2 can offer more promising therapeutic approaches for TBI patients.

In conclusion, this research not only provides novel scientific insights but also brings practical value to the clinical field. Through continued research and validation, we can potentially offer more effective treatment methods for TBI patients.

## Supplementary Information

Below is the link to the electronic supplementary material.

Supplementary file1 (JPG 961 KB) Construction of the TBI mouse model. Note: (A) Representative photographs of the whole brain from Sham and TBI mice at 1, 3, 7, and 14 days post-operation, white dashed lines indicate brain injury site; (B-D) mNSS scores (B) foot fault rates (C), and latency to fall in the rotarod test (D) of mice from Sham and TBI groups before and at 1, 3, 7, and 14 days after surgery; (E) Escape latency, swimming speed, and time to cross the platform in the MWM test of mice from Sham and TBI groups; (F) Swimming trajectory of mice from Sham and TBI groups; (G) Contusion volume in the ipsilateral cortex of mice at 3 days post-TBI (scale bar=1 mm); (H) Water content in the brain tissues of mice at 3 days post-TBI. * indicates difference from Sham group, p<0.05, *** indicates difference from Sham group, p<0.001, each group consisted of 8 mice

Supplementary file2 (JPG 484 KB) Expression of Key Factors and m6A Modification Levels in the Mouse Cortical Brain of the TBI Model. Note: (A) Protein expression of Wtap, Ythdf1, and Lcn2 in the cortical brain of Sham and TBI group mice detected by Western blot; (B) Quantification of m6A RNA methylation levels in the cortical brain of Sham and TBI group mice detected by m6A RNA methylation assay; (C) Detection of m6A modification levels in the cortical brain of Sham and TBI group mice by m6A dot plot experiment; (D) mRNA expression of Lcn2 in the cortical brain of Sham and TBI group mice detected by RT-qPCR; (E) m6A modification levels of Lcn2 in the cortical brain of Sham and TBI group mice detected by MeRIP qPCR. * indicates P<0.05 compared to the Sham group, with 8 mice in each group

Supplementary file3 (JPG 357 KB) Identification of Primary Cortical Neurons and Construction of an *in vitro* TBI Model. Note: (A) Immunofluorescence staining for the expression of β-III tubulin in primary cortical neurons; β-III tubulin: red fluorescence, nucleus (Hoechst33342): blue fluorescence staining (scale bar = 50 μm); (B) Measurement of LDH release in primary cortical neurons of each group; (C) Detection of apoptosis in primary cortical neurons of each group by flow cytometry. * indicates P<0.05 compared to the Control group, with the experiment repeated 3 times

Supplementary file4 (PDF 4156 KB) CRISPR/Cas9-Mediated Knockout of Wtap and Ythdf1 Genes in Primary Cortical Neurons. Note: (A) Plasmid structure for knockout of Wtap and Ythdf1 genes; (B-C) Validation of Wtap and Ythdf1 gene knockout by Sanger sequencing and PCR experiments; (D) Protein expression of Wtap and Ythdf1 in primary cortical neurons after Wtap or Ythdf1 knockout detected by Western blot. The experiment was repeated 3 times

Supplementary file5 (JPG 217 KB) Prediction of m6A Sites in the Lcn2 Gene. Note: (A) Prediction of m6A binding sites on the cDNA sequence of the Lcn2 gene using the SRAMP website; (B) Structural diagram of two highly confident m6A sites

Supplementary file6 (JPG 307 KB) Differential Analysis Results of Dataset GSE167459. Note: (A) Volcano plot of differentially expressed genes and heatmap of the top 50 differentially expressed genes in astrocytes of Sham and TBI group mice; (B) Volcano plot of differentially expressed genes and heatmap of the top 50 differentially expressed genes in microglia of Sham and TBI group mice; (C) Box plot of differential expression of Lcn2 in astrocytes and microglia of Sham and TBI group mice. Sham group: n=5, TBI group: n=5, * indicates P<0.05 compared to the Sham group

Supplementary file7 (JPG 1367 KB) Expression of Lcn2 in Cortical Neurons and Identification of Primary Glial Cells. Note: (A) Immunofluorescence staining for the proportion of Lcn2 and NeuN double-positive cells in the cortical brain of each group of mice (scale bar = 50 μm), * indicates P<0.05 compared to the Sham group, with 8 mice in each group; (B-C) Immunofluorescence staining (scale bar = 50 μm) and flow cytometry analysis for the specificity and purity of primary glial cells; (D) Immunofluorescence staining for the positive expression of GFAP in primary astrocytes (scale bar = 50 μm). The experiment was repeated 3 times

Supplementary file8 (JPG 261 KB) Activation of Glial Cells in Each Group. Note: (A) Immunofluorescence staining for the proportion of GFAP/C3-double positive cells in astrocytes of each group (scale bar = 50 μm); (B-C) Immunofluorescence staining for the proportion of M1-like and M2-like microglia in microglia of each group (scale bar = 50 μm). The experiment was repeated 3 times

Supplementary file9 (DOCX 16 KB)

## Data Availability

The datasets generated and analyzed during the current study are available from the corresponding author on reasonable request.
