# Supplementary figures and images for: Unraveling the molecular complexity: Wtap/Ythdf1 and Lcn2 in novel traumatic brain injury secondary injury mechanisms
Source: Cell Biol Toxicol. 2024 Aug 7;40(1):65. doi: 10.1007/s10565-024-09909-x (PMC11306654; doi:10.1007/s10565-024-09909-x)

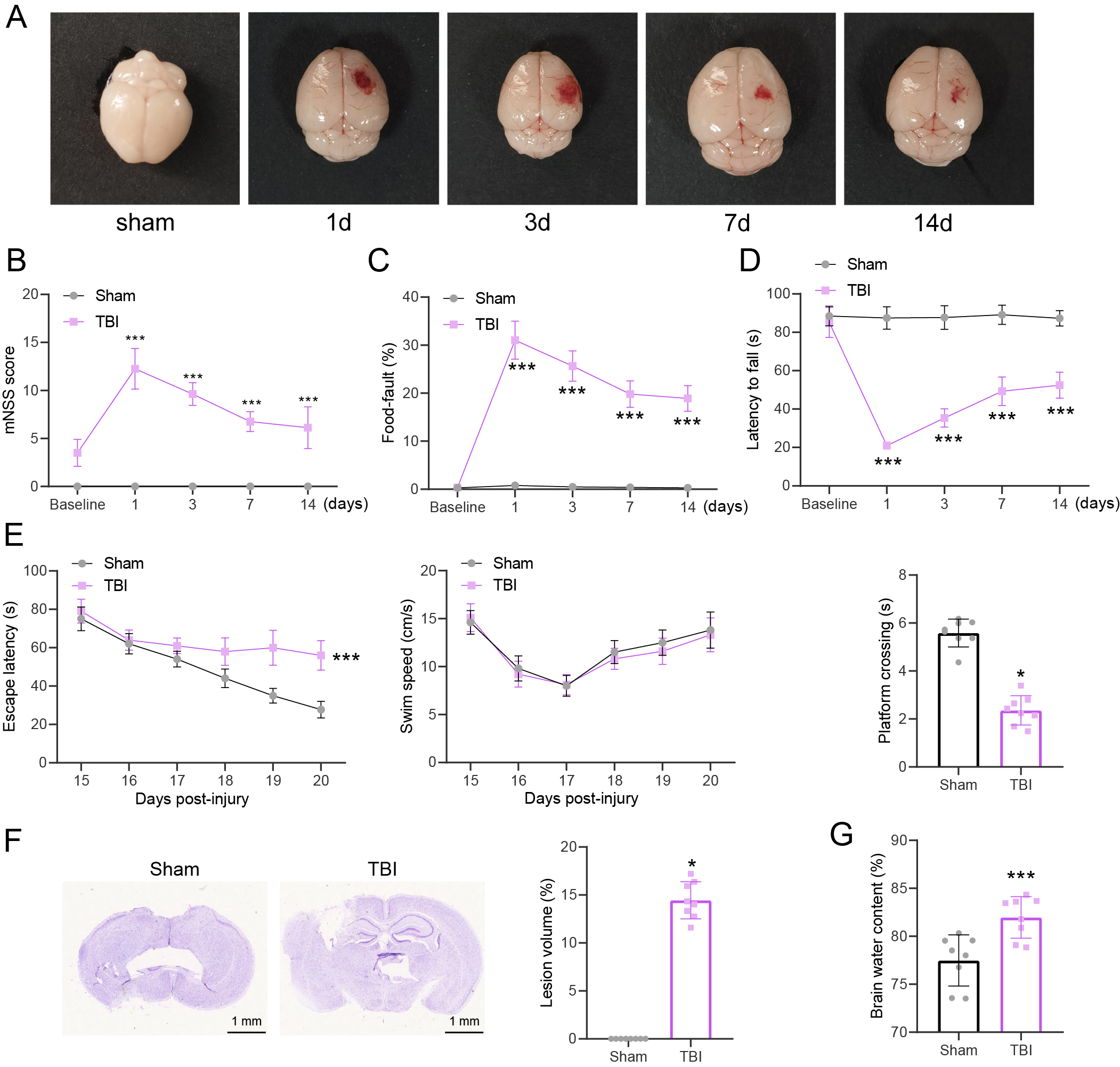

Supplement: Supplementary file 1 — Supplementary file1 (JPG 961 KB) Construction of the TBI mouse model. Note: (A) Representative photographs of the whole brain from Sham and TBI mice at 1, 3, 7, and 14 days post-operation, white dashed lines indicate brain injury site; (B-D) mNSS scores (B) foot fault rates (C), and latency to fall in the rotarod test (D) of mice from Sham and TBI groups before and at 1, 3, 7, and 14 days after surgery; (E) Escape latency, swimming speed, and time to cross the platform in the MWM test of mice from Sham and TBI groups; (F) Swimming trajectory of mice from Sham and TBI groups; (G) Contusion volume in the ipsilateral cortex of mice at 3 days post-TBI (scale bar=1 mm); (H) Water content in the brain tissues of mice at 3 days post-TBI. * indicates difference from Sham group, p<0.05, *** indicates difference from Sham group, p<0.001, each group consisted of 8 mice [file 10565_2024_9909_MOESM1_ESM.jpg]

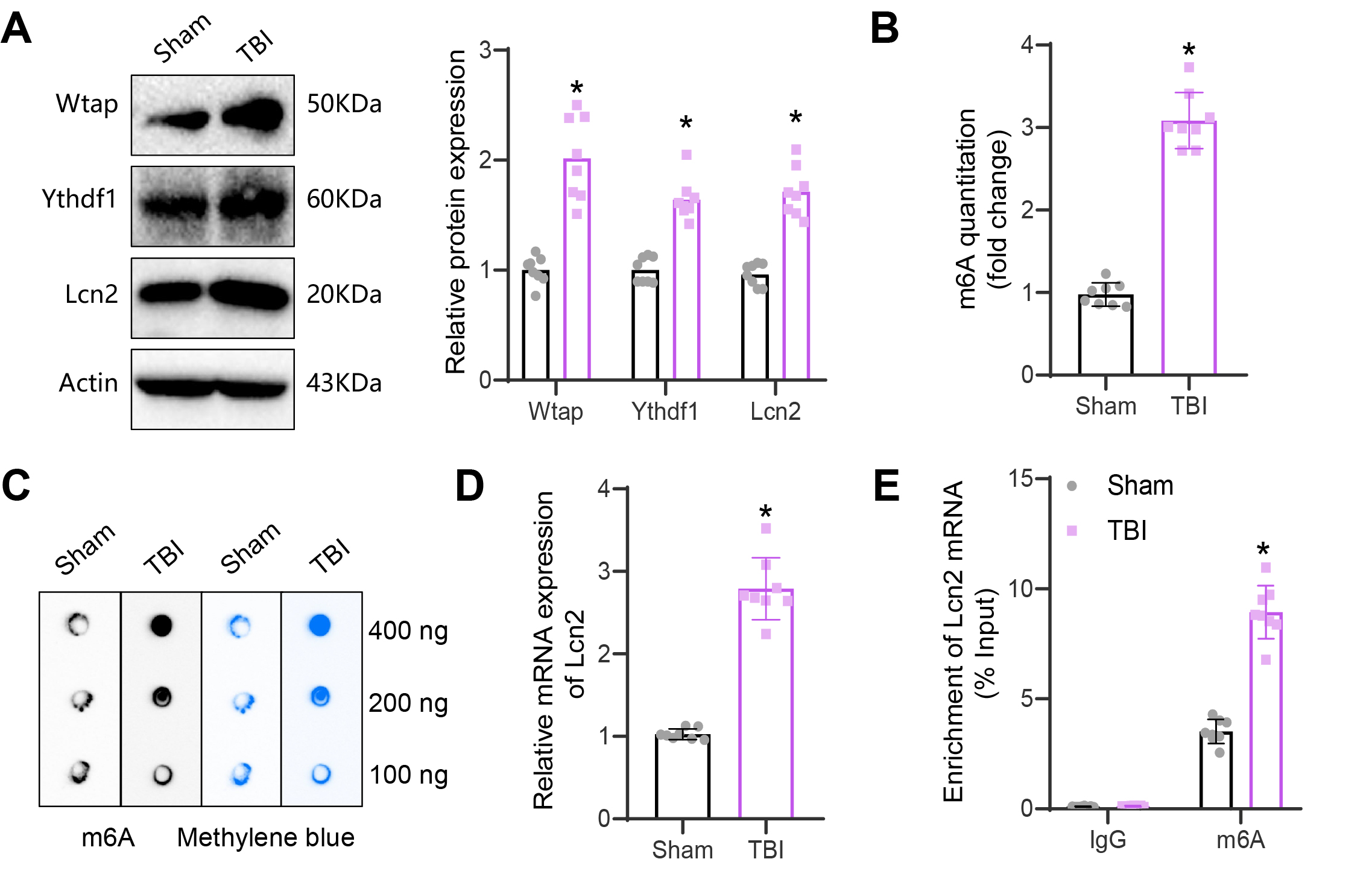

Supplement: Supplementary file 2 — Supplementary file2 (JPG 484 KB) Expression of Key Factors and m6A Modification Levels in the Mouse Cortical Brain of the TBI Model. Note: (A) Protein expression of Wtap, Ythdf1, and Lcn2 in the cortical brain of Sham and TBI group mice detected by Western blot; (B) Quantification of m6A RNA methylation levels in the cortical brain of Sham and TBI group mice detected by m6A RNA methylation assay; (C) Detection of m6A modification levels in the cortical brain of Sham and TBI group mice by m6A dot plot experiment; (D) mRNA expression of Lcn2 in the cortical brain of Sham and TBI group mice detected by RT-qPCR; (E) m6A modification levels of Lcn2 in the cortical brain of Sham and TBI group mice detected by MeRIP qPCR. * indicates P<0.05 compared to the Sham group, with 8 mice in each group [file 10565_2024_9909_MOESM2_ESM.jpg]

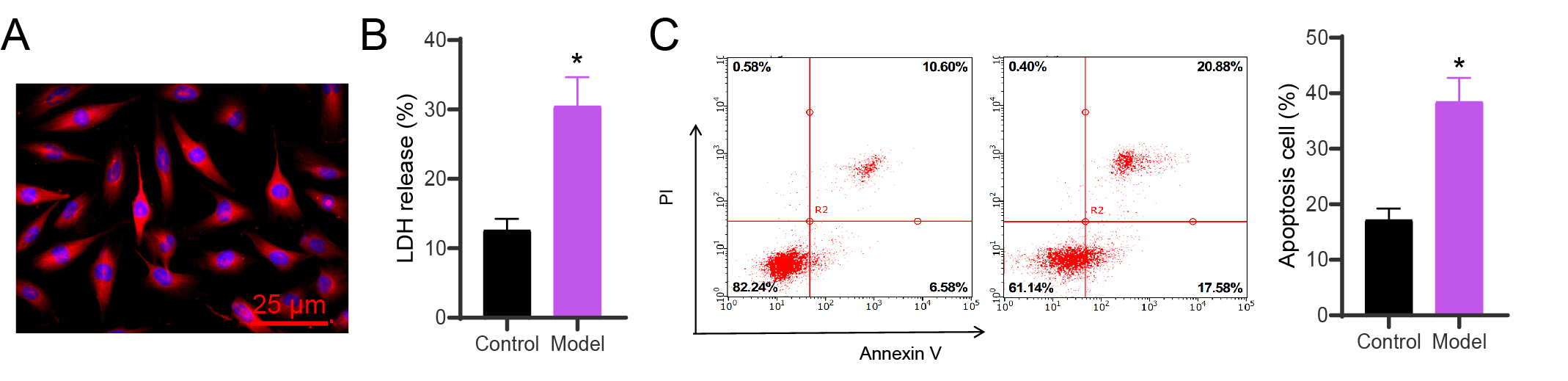

Supplement: Supplementary file 3 — Supplementary file3 (JPG 357 KB) Identification of Primary Cortical Neurons and Construction of an in vitro TBI Model. Note: (A) Immunofluorescence staining for the expression of β-III tubulin in primary cortical neurons; β-III tubulin: red fluorescence, nucleus (Hoechst33342): blue fluorescence staining (scale bar = 50 μm); (B) Measurement of LDH release in primary cortical neurons of each group; (C) Detection of apoptosis in primary cortical neurons of each group by flow cytometry. * indicates P<0.05 compared to the Control group, with the experiment repeated 3 times [file 10565_2024_9909_MOESM3_ESM.jpg]

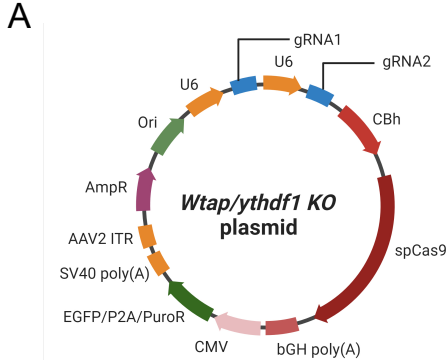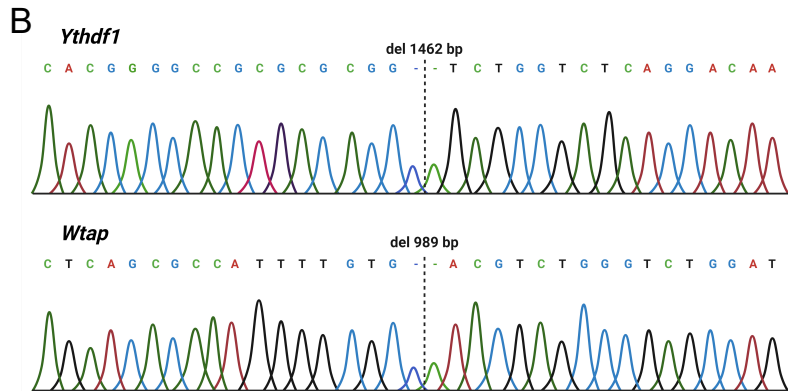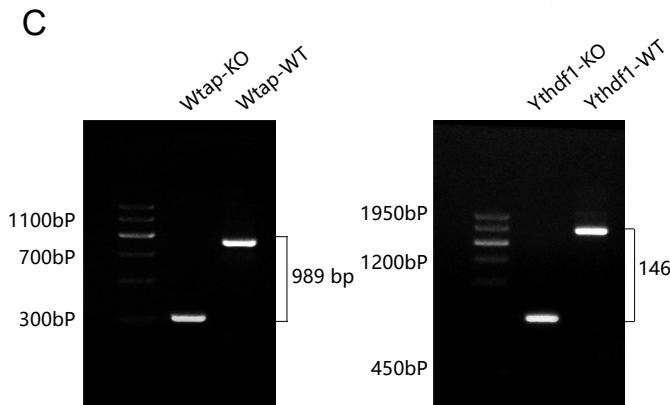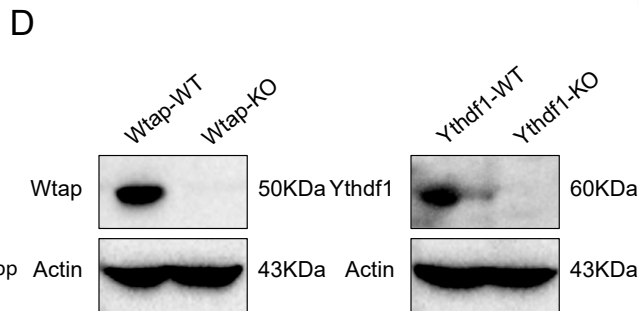

Supplement: Supplementary file 4 — Supplementary file4 (PDF 4156 KB) CRISPR/Cas9-Mediated Knockout of Wtap and Ythdf1 Genes in Primary Cortical Neurons. Note: (A) Plasmid structure for knockout of Wtap and Ythdf1 genes; (B-C) Validation of Wtap and Ythdf1 gene knockout by Sanger sequencing and PCR experiments; (D) Protein expression of Wtap and Ythdf1 in primary cortical neurons after Wtap or Ythdf1 knockout detected by Western blot. The experiment was repeated 3 times [file 10565_2024_9909_MOESM4_ESM.pdf]

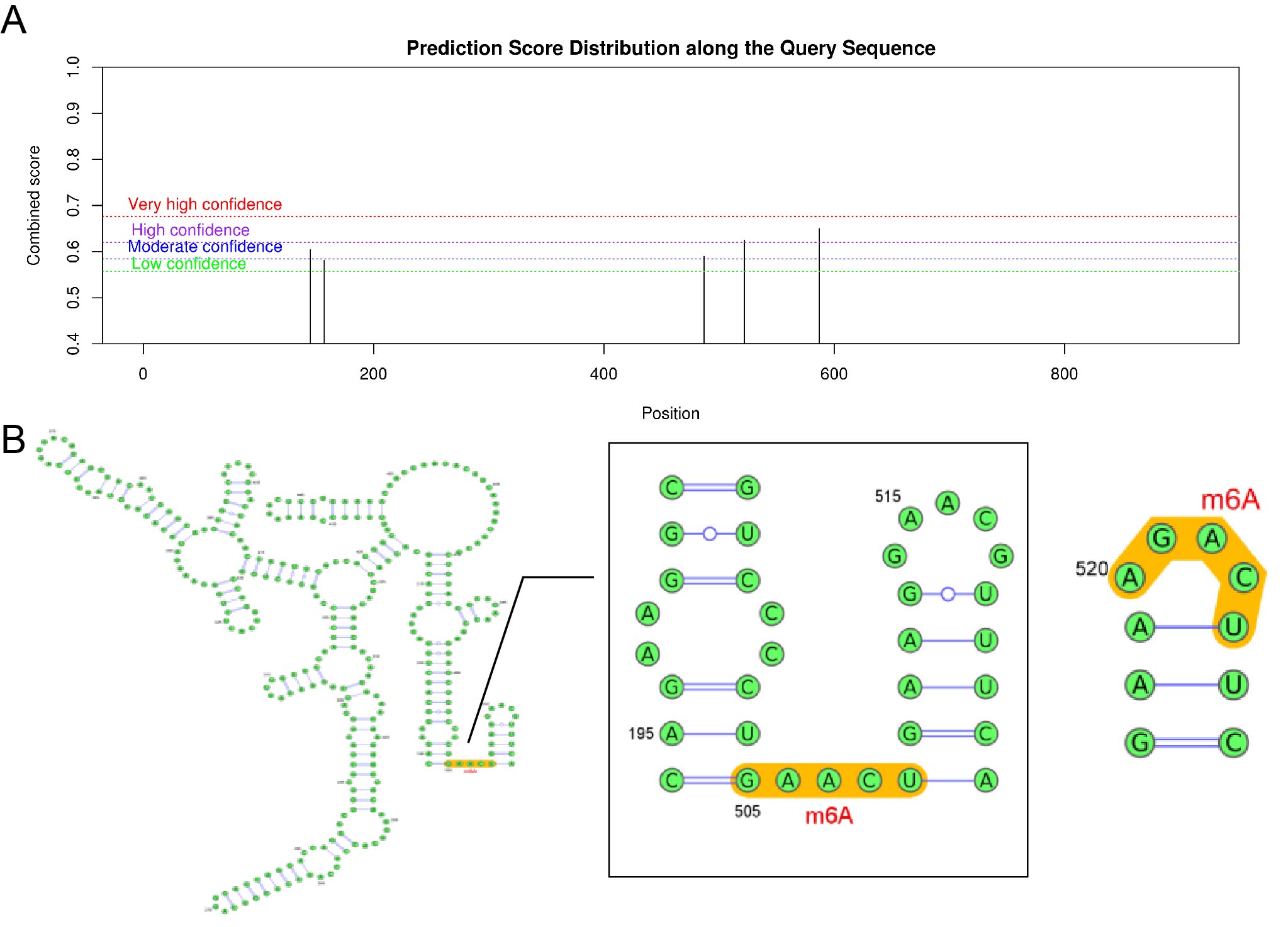

Supplement: Supplementary file 5 — Supplementary file5 (JPG 217 KB) Prediction of m6A Sites in the Lcn2 Gene. Note: (A) Prediction of m6A binding sites on the cDNA sequence of the Lcn2 gene using the SRAMP website; (B) Structural diagram of two highly confident m6A sites [file 10565_2024_9909_MOESM5_ESM.jpg]

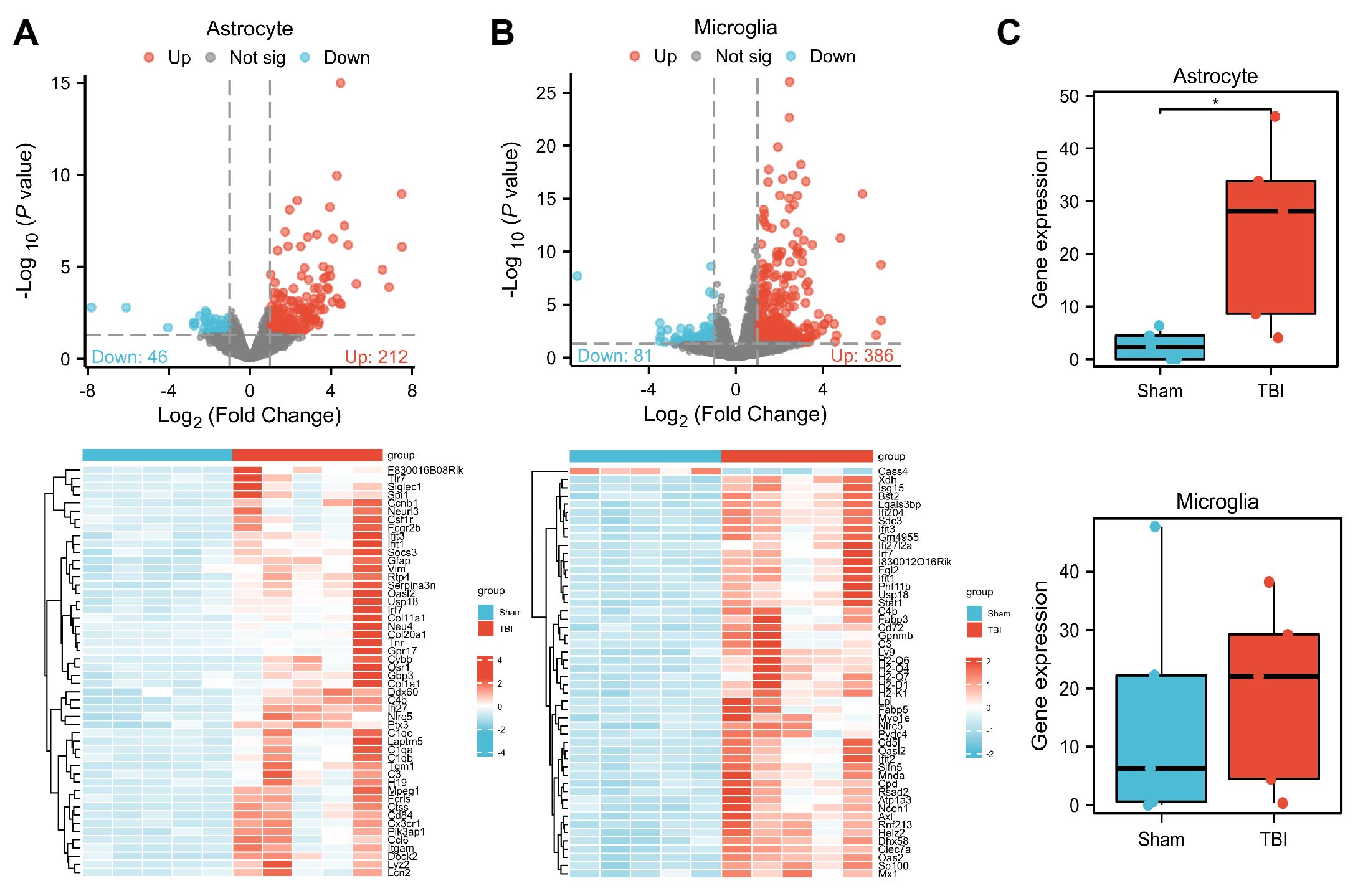

Supplement: Supplementary file 6 — Supplementary file6 (JPG 307 KB) Differential Analysis Results of Dataset GSE167459. Note: (A) Volcano plot of differentially expressed genes and heatmap of the top 50 differentially expressed genes in astrocytes of Sham and TBI group mice; (B) Volcano plot of differentially expressed genes and heatmap of the top 50 differentially expressed genes in microglia of Sham and TBI group mice; (C) Box plot of differential expression of Lcn2 in astrocytes and microglia of Sham and TBI group mice. Sham group: n=5, TBI group: n=5, * indicates P<0.05 compared to the Sham group [file 10565_2024_9909_MOESM6_ESM.jpg]

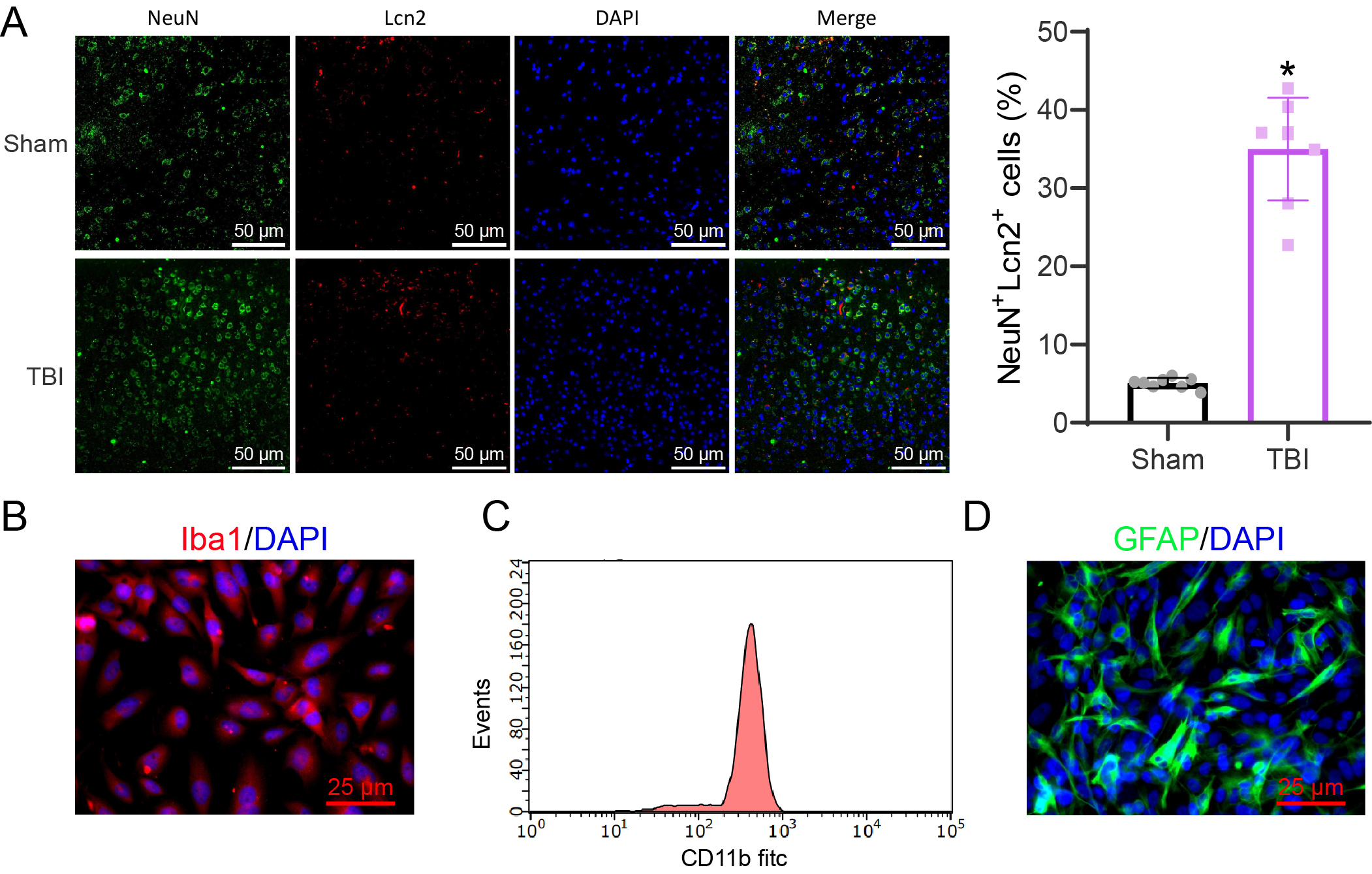

Supplement: Supplementary file 7 — Supplementary file7 (JPG 1367 KB) Expression of Lcn2 in Cortical Neurons and Identification of Primary Glial Cells. Note: (A) Immunofluorescence staining for the proportion of Lcn2 and NeuN double-positive cells in the cortical brain of each group of mice (scale bar = 50 μm), * indicates P<0.05 compared to the Sham group, with 8 mice in each group; (B-C) Immunofluorescence staining (scale bar = 50 μm) and flow cytometry analysis for the specificity and purity of primary glial cells; (D) Immunofluorescence staining for the positive expression of GFAP in primary astrocytes (scale bar = 50 μm). The experiment was repeated 3 times [file 10565_2024_9909_MOESM7_ESM.jpg]

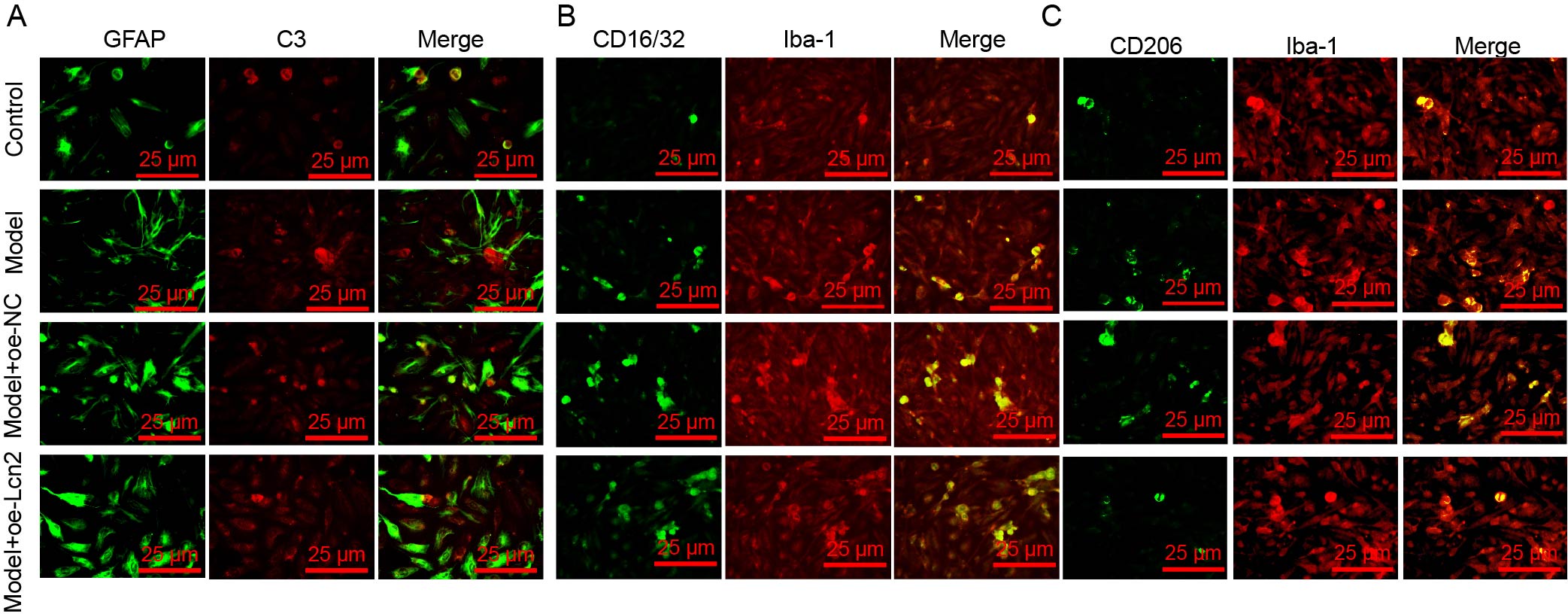

Supplement: Supplementary file 8 — Supplementary file8 (JPG 261 KB) Activation of Glial Cells in Each Group. Note: (A) Immunofluorescence staining for the proportion of GFAP/C3-double positive cells in astrocytes of each group (scale bar = 50 μm); (B-C) Immunofluorescence staining for the proportion of M1-like and M2-like microglia in microglia of each group (scale bar = 50 μm). The experiment was repeated 3 times [file 10565_2024_9909_MOESM8_ESM.jpg]
