# Supplementary material for: Unraveling the molecular complexity: Wtap/Ythdf1 and Lcn2 in novel traumatic brain injury secondary injury mechanisms
Source: Cell Biol Toxicol. 2024 Aug 7;40(1):65. doi: 10.1007/s10565-024-09909-x (PMC11306654; doi:10.1007/s10565-024-09909-x)
Supplement: Supplementary file 9 — Supplementary file9 (DOCX 16 KB) [file 10565_2024_9909_MOESM9_ESM.docx]

**Table S1. RT-qPCR primer sequences**

| Gene (mouse) | primer sequences |
| --- | --- |
| Lcn2 | Forward 5’-GGCCAGTTCACTCTGGGAAA-3’ |
|  | Reverse 5’-TGGCGAACTGGTTGTAGTCC-3’ |
| iNOS | Forward 5’-TCCTGGACATTACGACCCCT-3’ |
|  | Reverse 5’-AGGCCTCCAATCTCTGCCTA-3’ |
| TNF-α | Forward 5’-GTAGCCCACGTCGTAGCAAA-3’ |
|  | Reverse 5’-ACAAGGTACAACCCATCGGC-3’ |
| Amigo2 | Forward 5’-CGTAGGCACTTTAGCTCCGT-3’ |
|  | Reverse 5’-CGTGGATAAAGCCAAGTGCG-3’ |
| Serping1 | Forward 5’-AGTGCCCATGATGAGTAGCG-3’ |
|  | Reverse 5’-CACGGGTACCACGATCACAA-3’ |
| β-actin | Forward 5’-GATATCGCTGCGCTGGTCG-3’ |
|  | Reverse 5’-CATTCCCACCATCACACCCT-3’ |
